# Antimicrobial Gelatin Methacryloyl (GelMA)/Bioactive Glass Dental Adhesives

**DOI:** 10.3390/jfb17080394

**Published:** 2026-08-10

**Authors:** Tianyuan Zhao, Andrew M. Edwards, Adam D. Celiz, Julian R. Jones

**Affiliations:** 1 Department of Materials, Imperial College London, London SW7 2AZ, UK; tianyuan.zhao17@imperial.ac.uk; 2 Centre for Bacterial Resistance Biology, Imperial College London, London SW7 2AY, UK; a.edwards@imperial.ac.uk; 3 Department of Infectious Disease, Imperial College London, London SW7 2AY, UK; 4 Department of Bioengineering, Imperial College London, London SW7 2AZ, UK

**Keywords:** bioactive glass, GelMA adhesive, composite, dental remineralisation, antimicrobial activity

## Abstract

Oral diseases, such as periodontitis, remain a major global concern due to their high incidence and significant morbidity. The principal drivers are bacterial overgrowth and associated inflammation. Bioactive glass of the 45S5 Bioglass^®^ composition (BG) is known for its remineralising, osteogenic and antibacterial effects via ion release, but its application has been limited in oral environments. In this study, a photocurable GelMA hydrogel was developed as a carrier matrix for BG (1–15% *w*/*v*) to obtain synergy between the adhesive properties of the GelMA and the bioactivity of the glass. Incorporation of 10% *w*/*v* BG improved ultimate tensile strength (97 kPa) compared to pure GelMA (58 kPa) and reduced swelling by 18%. The composites showed ~60% higher adhesive strength on collagen sheets than GelMA alone. Tensile bonding strengths reached 54 kPa on collagen sheets and 23 kPa on tooth sections. Lap-shear adhesive strengths were 44 kPa on collagen and 18 kPa on implant metal. In vitro studies confirmed the composite’s biocompatibility with dental pulp stem cells and antibacterial activity against Escherichia coli and methicillin-resistant *Staphylococcus aureus*. Overall, the GelMA/BG composite presents a multifunctional platform for dental remineralisation with promising mechanical, adhesive and antibacterial performance.

## 1. Introduction

Periodontitis is a chronic inflammatory disease affecting the supporting structures of teeth, with a reported prevalence of approximately 47% among adults [1,2]. It is primarily initiated by bacterial colonisation of gingival tissues, which triggers host immune responses and the release of inflammatory mediators and matrix-degrading enzymes [3]. Among these, matrix metalloproteinases are upregulated, impairing new collagen formation and promoting degradation of existing collagen fibres within the periodontal connective tissue [4]. Prolonged inflammation leads to the breakdown of periodontal ligaments and resorption of alveolar bone, resulting in deep periodontal pocket formation [2]. Pathogenic bacteria colonise and grow in periodontal pockets, further promoting inflammation and limiting the antimicrobial efficacy of brushing techniques [3]. Mechanical plaque removal and systemic antibiotics are commonly prescribed in severe cases. However, antibiotic treatment faces limitations such as poor penetration into periodontal pockets, limited activity against biofilms, potential systemic side effects and the risk of promoting antibiotic resistance [5]. These challenges highlight the need for targeted, localised therapeutic strategies to be used alongside current approaches.

Bioactive glass (BG), exemplified by Bioglass^®^ 45S5 (45 wt% SiO_2_, 24.5 wt% Na_2_O, 24.5 wt% CaO and 6 wt% P_2_O_5_), has shown promise in dental applications [6]. For instance, microscale 45S5 glass particles added to toothpaste can alleviate dental hypersensitivity by forming hydroxycarbonate apatite (HCA) on exposed dentinal tubules [7]. 45S5 BG is extensively studied for its bioactivity, notably in promoting remineralisation and forming chemical bonds with native hard tissue. It has been shown to support dental pulp stem cell (DPSC) viability, metabolism and osteogenic differentiation through ion release as well as surface mineralisation processes [8,9]. BG activates osteogenic pathways by promoting expression of genes such as *RUNX2*, *DSPP* and *OCN*, and its dissolution ions have been shown to stimulate osteogenic differentiation of dental pulp stem cells (DPSCs) [10]. Also, the clinical success of BG in treating bone defects in patients suffering from osteomyelitis indicates antibacterial properties which could also combat periodontitis and peri-implantitis-related bacteria [11]. Antimicrobial properties are attributed to local pH modulation or ion release from the glasses [12]. However, the retention of BG in oral environments remains a challenge, as particles are easily removed by saliva, rinsing or toothbrushing. Thus, innovative delivery strategies, such as tissue adhesives, are required to enable non-invasive and localised application of BG, particularly on exposed dentin or within periodontal pockets, highlighting a significant unmet clinical need.

Hydrogels, inherently water-swollen polymeric networks, are well suited as soft biomimetic materials for the delivery of therapeutic particles for tissue regeneration due to their tuneable mechanical and tissue adhesive properties [13]. Gelatin methacryloyl (GelMA) hydrogels offer spatial and temporal control of photocrosslinking while preserving gelatin’s cell adhesion motifs enzymatic and biodegradability [14,15]. Furthermore, gelatin-derived hydrogels can serve as a mineralisation template, facilitating hydroxycarbonate apatite nucleation under biomimetic conditions [16]. GelMA’s stiffness and degradation rates can be tailored by adjusting its degree of modification (DoM) and polymer concentration [17]. Nichol et al. reported higher DoM and GelMA concentration enhanced compressive and tensile moduli while slowing enzymatic degradation by collagenases [15,18]. GelMA mechanical and adhesive properties are further influenced by crosslinking conditions, such as light intensity, exposure time and the types of photoinitiators [14,15]. Conventionally, ultraviolet (UV) light and type I photoinitiators, such as 1-[4-(2-hydroxyethoxy)-phenyl]-2-hydroxy-2-methyl-1-propane-1-one (I2959) [19] and lithium acylphosphinate [20], are used for GelMA crosslinking. Type I initiators absorb light energy and generate free radicals, propagating through MA groups to polymerise GelMA chains [21]. However, the utility of UV light is restricted in some medical applications, due to the potential risks of DNA damage. As a safer alternative, visible-light crosslinking using a ruthenium-based system, involving tris (2,2-bipyridyl)dichlororuthenium(II) hexahydrate (Ru) and sodium persulfate (SPS), has been employed [22]. Upon exposure to blue light (400–450 nm), Ru^2+^ is photoexcited and donates electrons to SPS, producing Ru^3+^ ions and sulfate radicals that initiate GelMA polymerisation [23]. In addition, these radicals may react with phenolic groups (e.g., tyrosine residues) on the gelatin backbone, enhancing network formation through secondary crosslinking. Ru^3+^ ions are eventually reduced back to Ru^2+^ ions, completing the catalytic cycle. Lim et al. demonstrated superior cytocompatibility of this Ru/SPS+ blue light system, compared to UV+ I2959, when breast adenocarcinoma cells were encapsulated within GelMA hydrogels; cell viability remained high with Ru/SPS, while I2959/UV led to a significant drop to ~50% at high doses [23].

GelMA hydrogels have also been shown to outperform fibrin glue as soft tissue sealants, offering nearly three-fold stronger adhesion on wet collagen-rich substrates [24]. The strong adhesion of GelMA comes from two main mechanisms: (1) the strong covalent bonding between methacryloyl groups and tissue thiol residues, wherein methacryloyl groups do not only crosslink gelatin chains but also undergo free radical-induced thiol-ene reactions with thiol groups [25], which are present in type I collagen and other extracellular matrix proteins [26]; (2) mechanical interlocking, whereby the uncrosslinked hydrogel solution infiltrates microgaps on substrate surfaces, then solidifies in situ upon light exposure, forming interpenetrating anchors at the tissue interface [27].

Building on these adhesive advantages, this project aims to develop an optimised GelMA/BG composite hydrogel adhesive for sealing periodontal pockets and implant–gingival tissue interfaces after mechanical removal of plaque. Addition of BG was intended to increase gel stiffness, deliver mineralising ions to teeth and impart antimicrobial properties. While these functions are well established in GelMA/BG systems through strategies such as mesoporous BG incorporation [28], BG compositional modification [29] and silanisation [30], previous work has primarily focused on optimising bulk properties rather than adhesive performance. In particular, most GelMA/BG systems have been developed as bulk hydrogels or 3D-printed scaffolds for bone regeneration [31,32], where mechanical integrity and bioactivity are prioritised over interfacial bonding. Moreover, these materials are typically photocrosslinked using UV light [28,33]. As a result, their behaviour as injectable or in situ forming adhesives under visible or blue light curing remains poorly understood. Critically, adhesion under wet conditions, especially to clinically relevant substrates such as collagen-rich soft tissues and dentine, has not been systematically investigated. In this study, we address these limitations by developing visible-light-curable GelMA/BG adhesive systems and evaluating how BG incorporation influences both bulk network properties and wet-tissue adhesion.

Our results demonstrate that the BG-infused hydrogel composite delivers both robust bonding to periodontal tissues and sustained antimicrobial ion release. Although bioactive glass has been incorporated into hydrogels for endodontic therapies and bone scaffolds in prior studies, its use specifically as a periodontal adhesive sealant remains largely unexplored. Thus, to our knowledge, this is one of the first studies to develop a BG-loaded, visible-light-cured hydrogel serving as a periodontal adhesive, showing both strong tissue adhesion and bioactive properties such as antimicrobial ion release and remineralisation potential. This combination of properties addresses a critical need in periodontal therapy: an adhesive material that can both seal the site and suppress local bacterial growth to support healing.

## 2. Materials and Methods

### 2.1. Bioactive Glass 45S5 Synthesis

To produce 100 g of bioactive glass (BG) 45S5 particles, 45 g of silicon dioxide (Prince’s Minerals, Stoke-on-Trent, UK), 43.8 g of calcium carbonate (Sigma-Aldrich, Dorset, UK, 471-34-1), 66.4 g of sodium carbonate (Sigma-Aldrich, 497-19-8) and 5.96 g of phosphorus pentoxide (Sigma-Aldrich, 1314-56-3) were mixed and melted in a PtAu5 crucible at 1400 °C for 1 h. The molten glass was then quenched in deionised (DI) water and dried at 120 °C for 2 days. The dried glass bulk was subsequently ball-milled into fine powder and sieved to collect particles smaller than 32 µm. Particle size distribution was measured using a Mastersizer 2000 laser diffraction analyser (Malvern Panalyticcal, Malvern, UK, Supplementary Figure S1). The amorphous structure of the synthesised BG was confirmed by X-ray diffraction (XRD), showing the absence of crystalline peaks (Supplementary Figure S2). To verify its apatite-forming ability, BG powders were immersed in Dulbecco’s Modified Eagle Medium (DMEM, Gibco, Paisley, UK, 61965-026) for 3 days and analysed for hydroxycarbonate apatite (HCA) formation by XRD (Supplementary Figure S3). XRD patterns were collected on a Bruker D2 Phaser diffractometer (Cu Kα, λ = 1.5406 Å) at 30 kV and 10 mA (Bruker AXS GmbH, Karlsruhe, Germany), scanning from 2θ = 20° to 60° with a 0.03° step size and no sample spinning.

### 2.2. Gelatin Methacryloyl Synthesis

Gelatin from porcine skin (Type A, 300 Bloom, Sigma-Aldrich, 9000-70-8) was methacrylated using methacrylic anhydride (MAA, Sigma-Aldrich, 760-93-0) following established protocols. Briefly, a 10% *w*/*v* gelatin solution was prepared by dissolving 10 g of gelatin in 100 mL of Dulbecco’s Phosphate-Buffered Saline (DPBS, Gibco, 14190-136) at 50 °C for 1 h. After complete dissolution, 6 g of MAA (0.6 g MAA per gram of gelatin) was added and stirred vigorously for 1 h. The resulting opaque solution was centrifuged at 4000 relative centrifugal force for 3 min. The transparent supernatant was collected and diluted with twice the volume of Milli-Q water to terminate the reaction. The GelMA solution was then purified using a 3.5 kDa MWCO dialysis membrane (Spectrum™, Repligen Corporation, Waltham, MA, USA, 132724) at 40 °C for 5–7 days, filtered through a 0.22 µm Steriflip filter (Merck, Felthan, UK, SCGP00525) for sterility, and freeze-dried for 3 days. GelMA derivatives were stored at −20 °C until further use.

Proton nuclear magnetic resonance (^1^H-NMR) spectroscopy with a 500 MHz instrument (AVANCE 500, Bruker BioSpin GmbH, Rheinstetten, Germany) was used to confirm the successful conjugation of methacryloyl groups to the gelatin backbone and to quantify the extent of modification (Supplementary Figure S4). For NMR analysis, 0.03% *w*/*v* of gelatin and freeze-dried GelMA were separately dissolved in deuterium oxide (D_2_O, Sigma-Aldrich, 7789-20-0) containing 0.75% *w*/*v* of 3-(trimethylsilyl) propionic-2,2,3,3-d4 (TMSP) acid as an internal reference. All samples were analysed at 37 °C. The amount of methacryloyl groups (AoM) was calculated using Equation (1):

(1) $$AoM\;(mmol/g)=\frac{\int Methacyloyl}{\int TMSP}\;\times \;\frac{9H}{1H}\;\times \frac{n(TMSP)\;[mmol]}{m\left(GelMA\right)\;[g]\;}$$

The total content of free primary amine groups in gelatin was quantified using a 2,4,6-trinitrobenzenesulfonic acid (TNBS, Sigma–Aldrich, 2508-18-2) assay. Gelatin samples (400 μg/mL) were prepared in 0.1 M sodium bicarbonate buffer (pH 8.5, Sigma–Aldrich, 497-19-8). A freshly prepared TNBS working solution (0.01% *w*/*v*) was mixed with samples at a 1:1 volume ratio and incubated at 37 °C for 2 h. The reaction was terminated by the addition of 10% *w*/*v* sodium dodecyl sulfate (SDS, Sigma-Aldrich, 151-21-3) and 1 M hydrochloric acid (HCl, Sigma-Aldrich, 76470-01-0). Glycine standards (0, 2, 4, 8 and 16 μg/mL) were used to generate a calibration curve. Absorbance was measured at 335 nm using a Clariostar Plus Microplate Reader (BMG Labtech GmbH, Ortenberg, Germany). The free primary amine content of gelatin was calculated from the standard curve and normalised to gelatin mass, yielding a value of 0.302 mmol/g. The DoM was calculated to be 63.4% by Equation (2):

(2) $$DoM=\frac{AoM}{total\;free\;amines\;in\;gelatin\;[mmol/g]}\;\times \;100\%$$

### 2.3. Fabrication of GelMA/BG Composites

Freeze-dried GelMA was dissolved in PBS at 40 °C to a concentration of 16.1% *w*/*v*. To prepare pure GelMA hydrogels, 3.5% *v*/*v* of tris (2,2-bipyridyl) dichlororuthenium (II) hexahydrate (Ru, Sigma-Aldrich, 50525-27-4) and 3.5% *v*/*v* of sodium persulfate (SPS, Sigma-Aldrich, 7775-27-1) were added to the solution to achieve a final GelMA concentration of 15% *w*/*v*. The solution was then loaded into silicone moulds and exposed to blue light (400–450 nm, 200 mW/cm^2^) for 360 s. Pure GelMA hydrogels without BG (denoted as 0BG) served as the control.

For GelMA/BG composites, a 16.1% *w*/*v* GelMA solution was prepared as above. Prior to adding photoinitiators, different concentrations of BG particles (1, 3, 5 and 10% *w*/*v*) were dispersed into the GelMA solution and stirred for 1 h. Then, 3.5% *v*/*v* each of Ru and SPS were added, followed by blue light exposure. “GelMA/xBG” refers to GelMA hydrogels containing x% *w*/*v* BG particles; for example, 1BG represents GelMA with 1% *w*/*v* BG.

### 2.4. Mechanical Characterisation

#### 2.4.1. Microscopic Morphology

The surface morphology and elemental composition of the hydrogel composites were examined using scanning electron microscopy (SEM) coupled with energy dispersive X-ray spectroscopy (SEM-EDX, JSM-6010LA, JEOL Ltd., Tokyo, Japan). GelMA hydrogels containing 1, 3, 5 and 10% *w*/*v* BG were fabricated as described previously. Pure GelMA hydrogels and BG particles alone were used as controls. All samples were freeze-dried to preserve internal structures and subsequently sputter-coated with a thin layer of gold nanoparticles to enhance conductivity. SEM imaging was conducted under an accelerating voltage of 20 kV and a working distance of 13 mm.

#### 2.4.2. Swelling Behaviour

To assess swelling behaviour, crosslinked composites containing 1, 3, 5 and 10% *w*/*v* BG were freeze-dried and weighed (w0). Samples were then immersed in 1 mL of DPBS containing 0.01% *w*/*v* sodium azide to prevent bacterial growth. The swollen weights (wt) were recorded at 1, 3 and 7 days. DPBS was replaced every three days. The swelling ratio (Δw) was calculated using Equation (3):

(3) $$\Delta w\left(\%\right)=\frac{wt-w0}{w0}\times 100\%$$

#### 2.4.3. Mechanical Tests

Pure GelMA and GelMA/BG solutions were mixed with Ru and SPS at a molar ratio of 1 mM:10 mM and crosslinked under blue light. Discs (8 mm diameter, 3.2 mm height) were fabricated for compression testing and dumbbell-shaped specimens (5 mm width, 14 mm length, 1.5 mm thickness) were fabricated for tensile testing. Universal compressive tests were conducted using an ElectroForce 5500 mechanical tester (TA Instruments, New Castle, DE, USA) with a 10 N load cell, applying a 1 mm/min compression rate until 60% strain. Tensile tests were performed using a Mach-1 Multiaxial Mechanical Tester (Biomomentum Inc., Laval, QC, Canada) with a 10 N load cell and the same loading rate. The compressive and elastic moduli were calculated from the linear region (0–5% strain) of the stress–strain curves.

#### 2.4.4. In Vitro Adhesion Assays

Adhesive performance was assessed using tensile and lap-shear tests on the ElectroForce 5500 mechanical testerwith a 10 N load cell. GelMA/BG precursor solutions containing photoinitiators were photocured onto wet collagen sheets that were affixed to glass slides using SuperGlue™ (Henkel AG & Co. KGaA, Düsseldorf, Germany). For tensile adhesion tests, cylindrical hydrogel specimens (8 mm diameter, 3 mm height) were subjected to uniaxial tension at 1 mm/min until failure and fracture modes were recorded.

To assess bonding to hard tissues, human dentine discs were prepared from caries-free molars sourced via the Imperial College Healthcare Tissue Bank (ICHTB; sub-collection R22003, London, UK), under ethical approval (17/WA/0161). Teeth were collected with informed anonymised consent and stored in 10% *v*/*v* formalin (Sigma-Aldrich, 50-00-0). Discs were polished with silicon carbide papers (P1000 and P4000) on a lapping machine (Kemet^®^ 4, Kemet International Ltd., Maidstone, Kent, UK) at 100 rpm, until a final thickness of 1.0 mm was achieved. The final discs were stored in 70% *v*/*v* ethanol (Sigma-Aldrich, 64-17-5). For tensile testing, the hydrogel precursor was photocured directly onto the polished dentine surface and the bonded construct was pulled in tension at 1 mm/min to measure adhesion strength. After failure, both the failed hydrogels and tooth discs were rinsed three times with DI water and freeze-dried for XRD analysis. Samples bonded to tooth sections were defined as T-0BG and T-10BG.

Lap-shear adhesion tests were conducted by applying 20 µL of precursor solution between two wet collagen sheets on glass slides, followed by photocuring. Samples were pulled in shear at 1 mm/min until detachment and adhesive area was used to calculate shear stress. Titanium alloy (Ti_6_Al_4_V, grade IV) substrates were prepared and tested using the same procedure.

### 2.5. In Vitro Cell Viability Assay

Dental pulp stem cells (DPSCs) were purchased from Lonza and expanded in Dulbecco’s Modified Eagle Medium (DMEM, Gibco, 61965-026) containing 10% *v*/*v* foetal bovine serum (Sigma-Aldrich, F9665) and 1% *v*/*v* penicillin–streptomycin (Gibco, 15140-122). Cells at passages P3–P9 were used for all experiments. DPSCs were detached using 0.25% trypsin–EDTA (Gibco, 25300-062), stained with 0.4% Trypan Blue (Gibco, 15250-061) and counted using an automated cell counter.

To evaluate the cytocompatibility of GelMA/BG hydrogels under indirect contact conditions, dental pulp stem cells were seeded in the bottom chamber of 24-well plates at a density of 30,000 cells in 700 µL of complete medium. In parallel, 150 µL of GelMA/BG precursor solution was photocrosslinked within Transwell^®^ inserts (Corning, 3422, Glendale, AZ, USA) to form hydrogel discs. To maintain hydrogel hydration, 300 µL of complete medium was added to the upper compartment of each insert. After 1-day incubation, live/dead staining was conducted using 1 µM Calcein AM (Merck, 206700) and 1 µM Propidium Iodide (Merck, S7109) prepared in DPBS. After 40 min of incubation at 37 °C in a humidified atmosphere with 5% CO_2_, cells were gently washed once with DPBS and imaged at 5× magnification. Positive and negative controls included Triton™ X-100 (Sigma-Aldrich, 9036-19-5)-treated and untreated DPSCs cultured on tissue culture plastic (TCP), respectively. Cell viability was calculated by Equation (4):

(4) $$cell\;viability\;\left(\%\right)=\frac{number\;of\;live\;cells\;}{number\;of\;total\;cells}\times \;100\%$$

For direct contact studies, 150 µL of GelMA/BG precursor solution was photocrosslinked in 48-well plates to form hydrogel substrates. DPSCs were seeded directly onto the hydrogels at 30,000 cells/mL (315 cells/mm^2^) and cultured at 37 °C, 5% CO_2_. Metabolic activity was assessed on days 1, 4, and 7 using the alamarBlue™ assay (Thermo Fisher, DAL1025, Dartford, UK) at a final concentration of 10% *v*/*v*. After a 4 h incubation, 100 µL of the supernatant was transferred to a black 96-well plate for fluorescence measurement using a Clariostar Plus Microplate Reader (excitation: 540–570 nm; emission: 580–610 nm), with fluorescence intensity expressed as relative fluorescence units (RFU). Wells containing only hydrogels (no cells) were included to subtract background signals.

At each time point, the remaining culture media were collected from each well, filtered through a 0.22 µm syringe filter and stored for subsequent ion release analysis. For inductively coupled plasma optical emission spectroscopy (ICP-OES, Thermo iCAP 6300, Thermo Fisher Scientific, Waltham, MA, USA), 0.5 mL of each medium sample was diluted with 9.5 mL of 2 M nitric acid to quantify Ca, Na, Si and P concentrations. Calibration standards were prepared at 0, 1, 2, 5, 10, 20 and 40 parts per million (ppm).

### 2.6. In Vitro Antimicrobial Assay

Mueller–Hinton Broth 2 (MHB2) medium was prepared by dissolving 8.8 g of MHB2 powder (Merck, 90922) in 400 mL of DI, followed by autoclaving for sterilisation. For MHB2 agar plates, 8.8 g of MHB2 powder and 6 g of bacteriological agar (VWR, 84609.0500, Lutterworth, UK) were dissolved in 400 mL of DI water. The solution was autoclaved and subsequently poured into 10 mm Petri dishes (20 mL per plate). Plates were allowed to solidify at room temperature and stored at 4 °C for colony-forming unit (CFU) counting experiments.

*Escherichia coli* (*E. coli*, MG1665) and methicillin-resistant *Staphylococcus aureus* (MRSA, JE2) were thawed from storage at −70 °C in 15% glycerol and cultured in 3 mL of MHB at 37 °C overnight in a shaker (180 rpm), respectively. Each culture was diluted 10,000-fold by adding 1 µL to 10 mL of fresh MHB2. To determine the initial seeding density, 20 µL of the diluted suspension was serially diluted (10^−1^ to 10^−6^) in PBS, then 10 µL of each dilution was plated on MHB2 agar. After 1-day incubation at 37 °C, colonies were counted and used to calculate initial bacterial density.

To evaluate the antibacterial efficacy of GelMA/BG hydrogels, 100 µL of pre-formed hydrogel sample was placed in 96-well plates and incubated with 100 µL of bacterial suspension. TCP was used as the bacterial growth control, 0BG served as the BG-free control and MHB2 alone was used as the negative control. After 1 and 2 days of incubation, 20 µL of each bacterial supernatant was serially diluted in PBS (10^−1^ to 10^−6^). From each dilution, 10 µL was plated onto MHB2 agar plates and incubated for CFU counting.

To directly compare the antibacterial effects of dry BG particles versus BG embedded within hydrogels, BG suspensions were prepared at 3 and 10% *w*/*v* by dispersing 30 mg or 100 mg of BG particles in 1 mL of PBS. Each suspension was mixed with 100 µL of bacterial culture. Similarly, 100 µL of 3BG or 10BG hydrogel was incubated with 100 µL of bacterial suspension. After 1 day, samples were serially diluted and plated for bacterial CFU quantification.

### 2.7. Statistical Analysis

All quantitative data are presented as the mean ± standard deviation, with a minimum sample size of *n* ≥ 3 for all groups. Statistical analyses were performed using GraphPad Prism 10.0. Data normality was evaluated using the Shapiro–Wilk test. For datasets that followed a normal distribution, parametric tests were applied, including unpaired *t*-tests, one-way ANOVA and two-way ANOVA, followed by Tukey’s multiple comparisons test where appropriate. For datasets that did not meet normality assumptions, non-parametric Kruskal–Wallis tests were used for comparisons across multiple groups. Statistical significance was defined as follows: *p* > 0.05 (not significant, n.s.), *p* < 0.05 (*), *p* < 0.01 (**) and *p* < 0.001 (***) and *p* < 0.0001 (****).

## 3. Results

### 3.1. Physical Characterisation of GelMA/BG Composites

**Figure 1:**
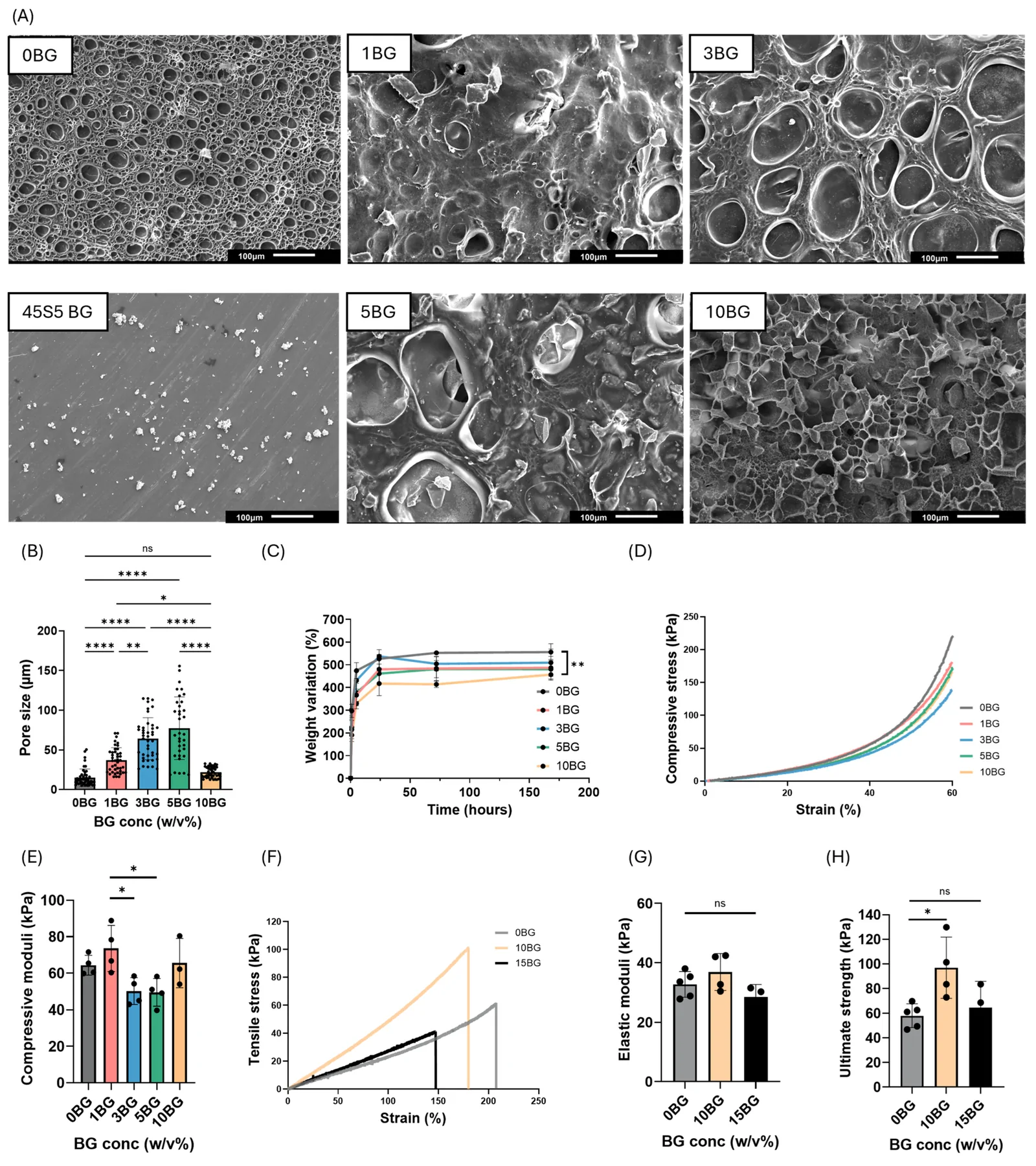
Morphological and mechanical characterisation of GelMA/BG composite with 0–15% *w*/*v* BG. (**A**) SEM micrographs showed the microscale morphologies of lyophilised GelMA/BG hydrogels. (**B**) Average pore size quantified from SEM images using ImageJ 1.53t, plotted as a function of BG concentration (% *w*/*v*). (**C**) Weight change (%) as a function of immersion time in PBS at 37 °C, indicating water uptake behaviour. (**D**) Representative compressive stress–strain curves up to 60% strain. (**E**) Compressive moduli of adhesives derived from the initial linear region (0–5% strain). (**F**) Representative tensile stress–strain curves of photocrosslinked hydrogel strips. (**G**) Elastic moduli calculated from the linear region of the tensile stress–strain curves. (**H**) Ultimate tensile strengths of GelMA/BG composites, showing the maximum stress before fracture. Data are presented as the mean ± standard deviation (*n* ≥ 3); not significant (n.s., *p* > 0.05), * (*p* < 0.05), ** (*p* < 0.01), **** (*p* < 0.0001).

Under SEM, the lyophilised GelMA hydrogel exhibited its characteristic, interconnected porous structure, with a broad distribution of pore sizes ranging from 5.2 to 50.5 µm, where pore size refers to the equivalent diameter of the enclosed void spaces within the polymer network [34], and mean equivalent pore diameters of 15.1 ± 11.0 μm (Figure 1A,B). BG 45S5 particles (Figure 1A) had a modal particle size of 14.5 μm (Supplementary Figure S1). Incorporation of BG at varying concentrations (1, 3, 5 and 10% *w*/*v*) into the GelMA matrix resulted in alterations in pore size. Compared to the control (0BG), the mean pore sizes increased to 37.1 ± 15.7 μm, 64.1 ± 26.4 μm and 67.0 ± 42.8 μm, respectively. Interestingly, the 10BG group exhibited a significantly smaller pore size (21.8 ± 6.7 μm) than other BG-containing formulations, potentially indicating a more dense or more stable microstructure. Elemental mapping of silicon confirmed the homogeneous dispersion of BG particles within the GelMA matrix (Supplementary Figure S5).

The swelling ratios of GelMA/BG hydrogels were evaluated after 1, 3 and 7 days of incubation in PBS at 37 °C (Figure 1C). After 7 days, the control (0BG) showed a 556% ± 37% increase in weight. Incorporation of BG significantly reduced swelling, with an increase in weight of 487% ± 50% (1BG), 509% ± 60% (3BG), 480% ± 43% (5BG) and 457% ± 25% (10BG). Among these, 10BG exhibited the most pronounced reduction. This trend in swelling behaviour was consistent with the porous structures observed under SEM (Figure 1A), where higher BG concentration appeared to limit water uptake.

Compressive testing of freshly photocrosslinked hydrogels showed that introducing BG did not significantly affect the compressive moduli (0BG: 64.3 ± 5.5 kPa vs. 10BG: 65.5 ± 13.5 kPa) until the strain reached 60%, indicating the structural stability of crosslinked networks (Figure 1D,E). Considering that oral adhesives are likely to undergo tensile forces in use, tensile tests were also performed (Figure 1F–H). Likewise, there were no significant differences in elastic moduli among three formulations, although a slight increase from 32.7 ± 4.3 kPa to 36.9 ± 6.2 was discovered by adding 10% *w*/*v* BG (Figure 1G). However, increasing glass content to 10BG did increase the ultimate strength to 96.8 ± 24.9 kPa (Figure 1H), compared to 0BG (58.0 ± 9.5 kPa). To determine whether increasing the glass content further would increase the tensile strength, 15BG composites were produced and tested, but the tensile strength reduced to 64.5 ± 21.4 kPa. The mechanical studies suggested that 10BG might be the optimal concentration as the glass did not interfere with GelMA crosslinking, but also greatly enhanced the ultimate strength of the composites under tension forces.

### 3.2. Tensile and Lap-Shear Adhesion Characterisations of GelMA/BG Adhesives

**Figure 2:**
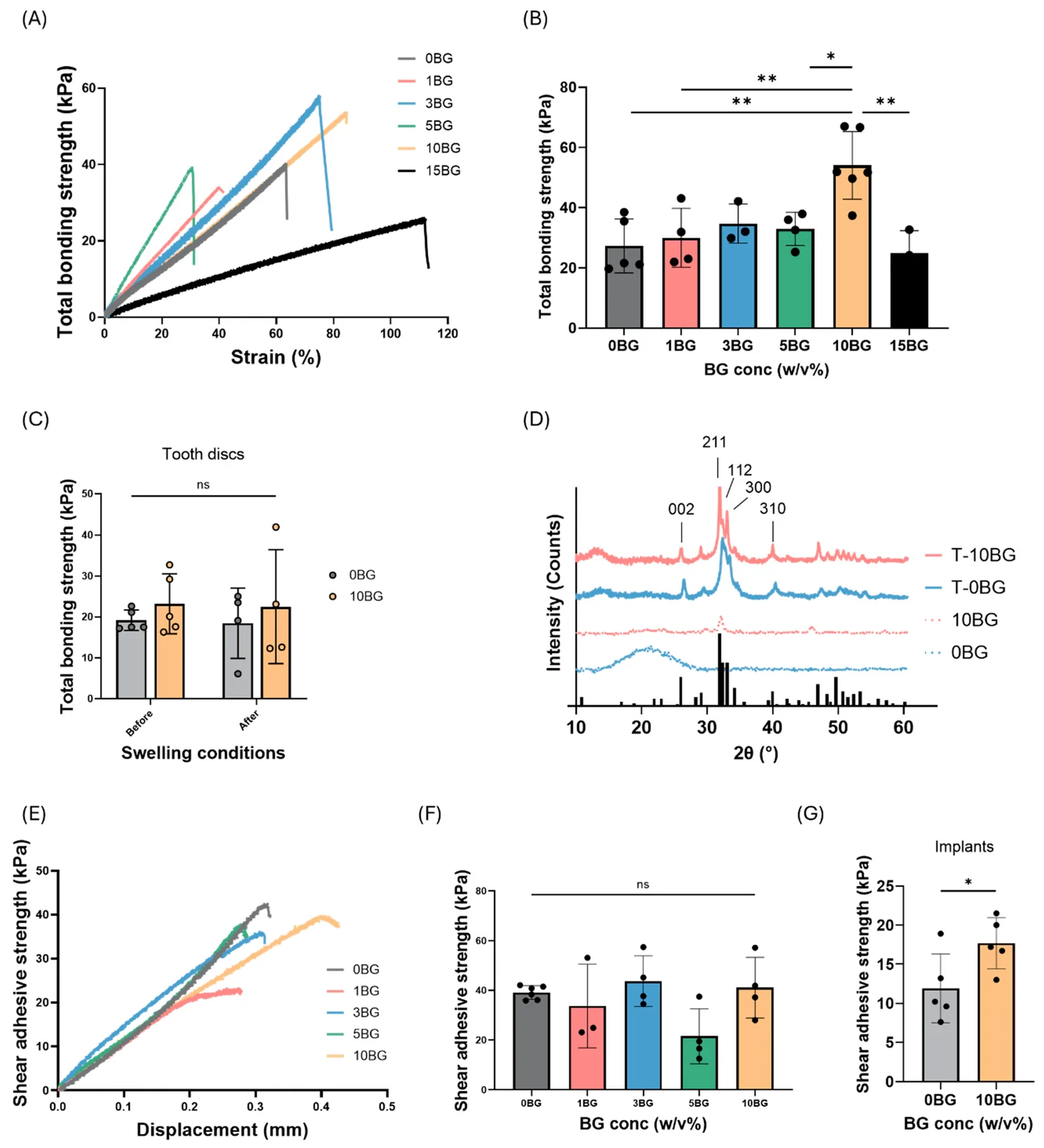
In vitro adhesive properties of GelMA/BG composite adhesives with 0, 1, 3, 5 and 10% *w*/*v* BG. Precursor solutions were loaded onto wet collagen sheets and photocrosslinked into cylindrical specimens (6 mm diameter and 3.2 mm height). (**A**) Representative tensile adhesion curves on wet collagen sheets. (**B**) Maximum tensile bonding strength on collagen sheets for different BG concentrations. (**C**) Maximum tensile adhesion to human tooth discs immediately after photocuring and after 7 days in DMEM. (**D**) XRD patterns of 0BG and 10BG adhesives, either alone or bonded to tooth sections (denoted as T-0BG and T-10BG), following 7-day incubation in DMEM, showing hydroxycarbonate apatite formation (ICCD reference code: 00-009-0432) [35]. (**E**) Representative lap-shear adhesion curves on collagen substrates. (**F**) Lap-shear strength of GelMA/BG adhesives bonded between collagen sheets. (**G**) Lap-shear strength of GelMA/BG adhesives on titanium alloy (Ti–6Al–4V) plates. Data are presented as the mean ± standard deviation (*n* ≥ 3); not significant (n.s., *p* > 0.05), * (*p* < 0.05), ** (*p* < 0.01).

Hydrogel precursor solutions were loaded onto wet collagen sheets, mimicking oral soft tissues, and photocrosslinked as cylinders under visible light (Figure 2A,B). Vertical tensile force was applied to the adhesive and substrate; the strain and the total bonding strength to collagen were recorded until failure. Significantly higher bonding strength was exhibited by 10BG samples than other samples. Specifically, compared to 0BG (27.3 ± 9.0 kPa), the bonding strength doubled with the addition of 10% *w*/*v* BG (54.1 ± 11.3 kPa). Lower concentrations of BG did not cause significantly increased bonding strength compared to 0BG. An excessive amount of BG (15% *w*/*v*) led to a lower adhesive strength of 24.9 ± 7.5 kPa and a shift from cohesive to adhesive failure, likely due to network disruption. Further testing on 15BG was therefore not carried out.

Considering oral applications, adhesion to human tooth sections was evaluated both immediately after photocuring and following 7-day incubation in fully supplemented DMEM (Figure 2C). Initially, all fresh hydrogel samples exhibited weaker adhesion to tooth surfaces compared to collagen sheets, with no significant differences observed between 0BG and 10BG (19.2 ± 2.5 kPa and 23.2 ± 7.3 kPa, respectively). Also, the failure mode of all tested compositions shifted from cohesive failure within the hydrogel, when tested with the collagen, to adhesive failure at the hydrogel–tooth interface. After 7 days of incubation, total adhesive strength to teeth remained stable (22.5 ± 3.1 kPa for 0BG and 22.5 ± 13.9 kPa for 10BG). XRD analysis confirmed that 0BG remained amorphous over time and revealed distinct hydroxycarbonate apatite (HCA, ICCD reference code: 00-009-0432) peaks in 10BG composites (Figure 2D) [35]. Notably, tooth samples adhered with 10BG hydrogels and displayed more prominent HCA peaks than those with 0BG, suggesting BG-mediated interfacial mineralisation during incubation. These findings indicate that while bulk adhesion remains stable, the presence of BG promotes bioactive integration at the tooth interface, potentially enhancing long-term bonding performance.

Lap-shear adhesion tests were also performed to investigate the adhesive performance of the composites under shear forces (Figure 2E,F). A total of 20 μL of hydrogel precursor solution was pipetted between pieces of wet collagen sheets, followed by photocuring. A thin layer of yellow adhesive was visible between collagen sheets, allowing measurements of adhesion area. Different from the tensile adhesion tests, incorporation of BG particles did not dramatically enhance the lap-shear adhesive strength, with values ranging from 33.7 ± 16.8 kPa to 43.7 ± 10.2 kPa.

The adhesive strength to titanium-based Ti6Al4V alloys was also tested (Figure 2G). The adhesives exhibited much lower adhesion properties to metal plates than to collagen sheets, with the control group (0BG) showing an adhesive strength of 11.9 ± 4.4 kPa. Interestingly, the addition of 10% *w*/*v* BG improved the lap-shear adhesive strength to dental implant metals, increasing it to 17.7 ± 3.3 kPa, suggesting improved compatibility and interfacial bonding with metallic surfaces.

### 3.3. In Vitro Cytotoxicity Assays of GelMA/BG Composites

**Figure 3:**
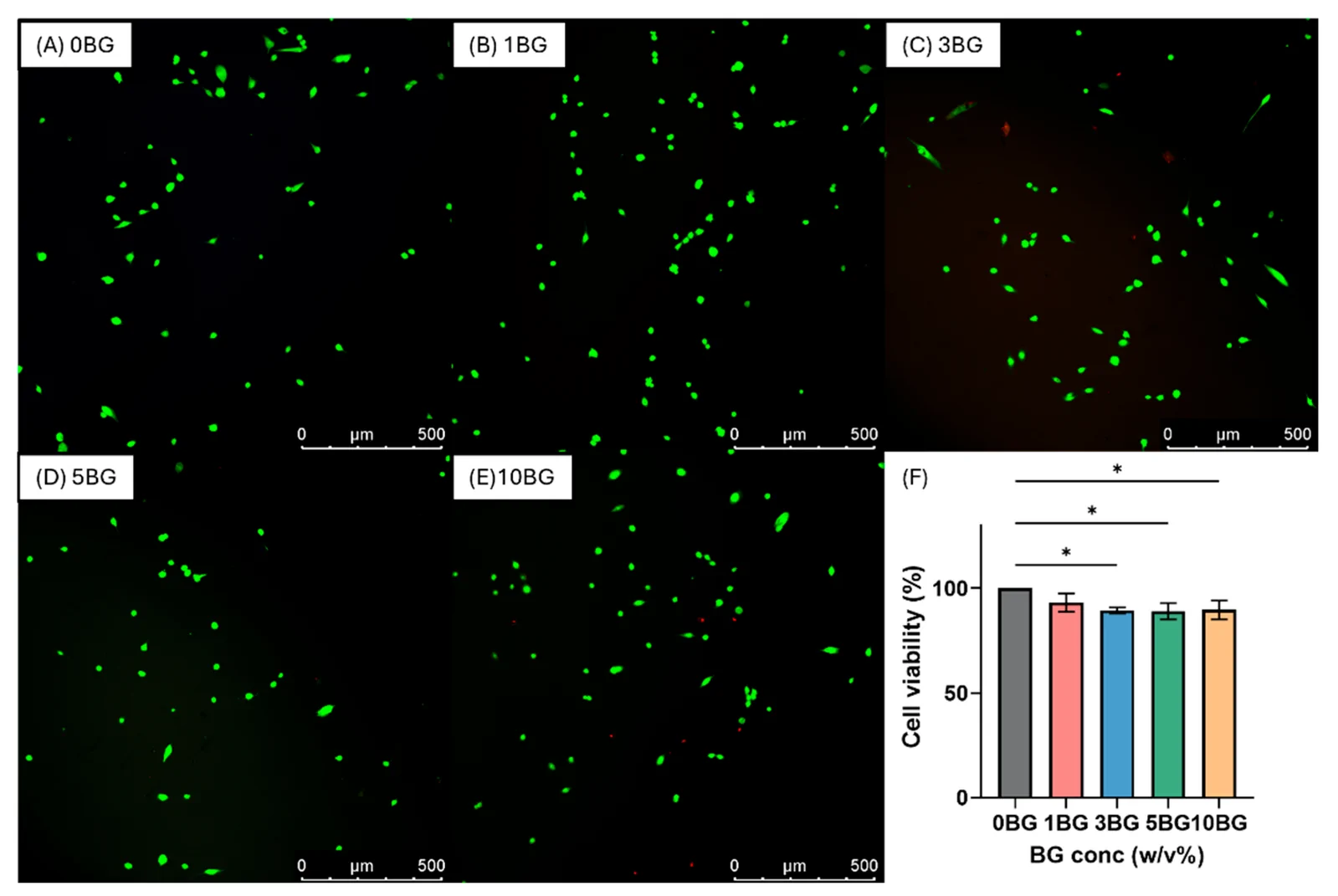
In vitro 2D seeding of DPSCs in indirect contact with GelMA/BG adhesive composites with varying BG concentrations. (**A**–**E**) Representative fluorescence images of live (green)/dead (red) stained DPSCs after 1 day of indirect contact with hydrogels in Transwell^®^ inserts (scale bar = 500 µm). (**F**) Quantitative viability analysis based on fluorescence imaging, expressed as the percentage of viable cells relative to total cells. Data are presented as the mean ± standard deviation (*n* ≥ 3); * (*p* < 0.05).

To study the influence of BG dissolution products on DPSCs, which were used as model cells. DPSCs were cultured indirectly with the hydrogels, so that the cells were only exposed to the dissolution products of the gels. The gels were fabricated in the Transwell^®^ inserts, while the DPSCs were cultured in the base of the well plates. DPSC morphologies were observed by confocal microscopy after 1 day of culture (Figure 3A–E). Green fluorescence was observed in viable cells, while dead cells stained red. High cell viability was observed across all samples. Control cells appeared well adhered to TCP with elongated morphologies, which is indicative of a healthy DPSC phenotype. Quantitative analysis revealed that the cells cultured with the BG-containing hydrogels showed a slight reduction in cell viability, with values of 93.1 ± 4.4% (1BG), 89.3 ± 1.4% (3BG), 89.0 ± 3.9% (5BG) and 89.6 ± 4.4% (10BG) (Figure 3F). DPSC viability was greater than 85% in the dissolution products from all GelMA/BG composites, indicating the non-toxic behaviour under indirect contact. According to ISO-10993-5/12, 70% relative cell viability is considered non-toxic [36,37].

**Figure 4:**
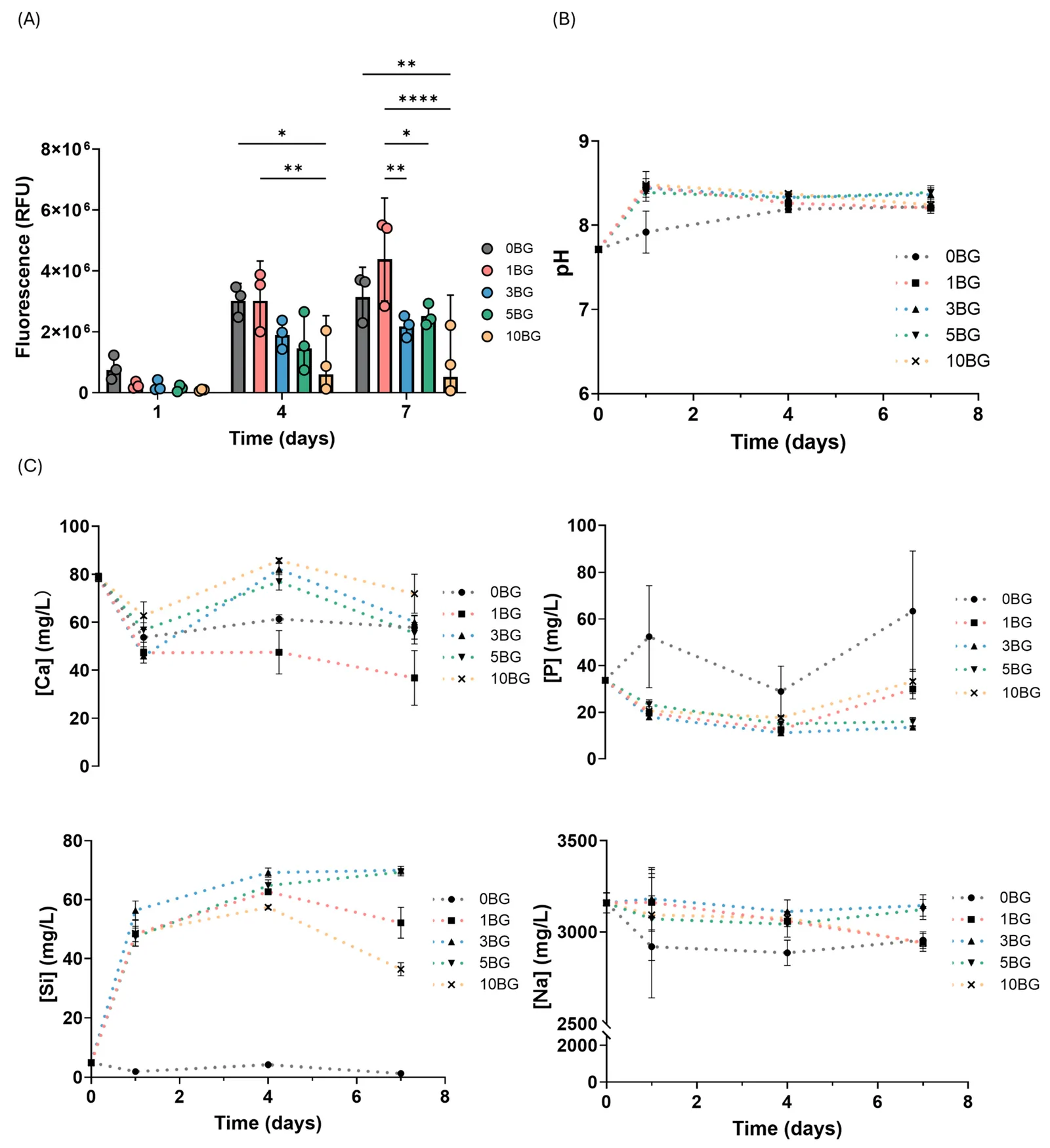
In vitro 2D seeding of DPSCs on GelMA/BG adhesive composites of varying BG concentrations. (**A**) Metabolic activity of DPSCs over 7 days, measured using a 10% *v*/*v* alamarBlue assay and expressed as RFU. (**B**) pH changes in culture medium over time, reflecting local environmental conditions induced by BG dissolution. (**C**) Elemental profiles of Ca, Si, P and Na in cell culture media from GelMA/BG adhesives collected before each media change and measured using ICP-OES. Data are presented as the mean ± standard deviation (*n* ≥ 3); not significant (*p* > 0.05), * (*p* < 0.05), ** (*p* < 0.01), **** (*p* < 0.0001).

Following the indirect cytotoxicity assays, DPSCs were top seeded directly on hydrogel discs containing varying BG concentrations. The metabolic activity of cultured DPSCs was then measured over 7 days (Figure 4A). On day 1, all BG-containing groups showed lower metabolic activity compared to 0BG (813,243 ± 397,655 RFU), with values ranging from 247,570 ± 113,515 RFU (1BG) to just 89,663 ± 30,747 RFU (10BG), suggesting an initial inhibitory effect of BG on DPSC viability or metabolic function. This is common for bioactive glasses in vitro [8]. By day 4, all groups showed a clear increase in metabolic activity. The 1BG group showed slightly higher metabolic activity than 0BG, while smaller increases were observed for 3BG and 5BG, reaching 1,932,147 ± 476,793 RFU and 1,647,230 ± 961,201 RFU, respectively. The 10BG group also increased to 1,012,835 ± 965,560 RFU but remained lower than the other groups, suggesting a sustained inhibitory effect at the highest BG concentration. By day 7, cells on 1BG exhibited the highest metabolic activity (4,586,111 ± 1,508,789 RFU), suggesting enhanced proliferation over time. The 3BG and 5BG groups also showed increased metabolic activity, although both remained lower than 0BG. The 10BG group remained the lowest, at approximately 1,070,000 RFU, confirming a dose-dependent inhibitory effect at high BG content. Importantly, metabolic activity increased over the 7-day culture period across all formulations. These findings indicate that while high concentrations of BG can impair DPSC activity, low concentrations, specifically 1BG, may promote sustained cell proliferation and metabolic health over time.

To understand the biological responses observed in DPSC cultures, patterns of pH changes and ion release from GelMA/BG composites were investigated in the presence of DPSCs over 7 days, with the cell culture medium changed on days 1, 4 and 7 (Figure 4B,C). The initial pH of fresh medium was approximately 7.7. After incubation, BG-containing groups exhibited an increase in pH compared to 0BG. On day 1, 1BG, 3BG, 5BG and 10BG reached pH values of 8.1 ± 0.1, 8.1 ± 0.1, 8.3 ± 0.1 and 8.2 ± 0.1, respectively, whereas 0BG remained closer to physiological levels (7.9 ± 0.3). This elevated pH persisted over 7 days.

The alkalinity is consistent with Ca^2+^ and Na^+^ ion exchange with H^+^ from the media. In all media, calcium levels were initially maintained at a high level (~80 mg/L). After 1 day, calcium concentrations decreased across all groups. Following media changes, Ca concentrations in 0BG and 1BG samples stabilised at 40–60 mg/L. In contrast, 3BG, 5BG and 10BG samples showed increases in Ca concentration to 80–100 mg/L by day 4, indicating higher net calcium release from higher BG loadings. Media containing 0BG showed negligible Si content, verifying that BG is the only source of Si. For all BG groups, Si levels rose sharply during the first day, stabilising at 60–70 mg/L thereafter. The measured Si levels on day 4 appeared lower, after media changes, reflecting dilution due to fresh medium rather than cessation of ion release. Cumulative elemental release from the composites is shown in Supplementary Figure S6. All BG groups continued to release Si species, although at different release rates. P levels were highest in the 0BG group, while BG-encapsulating groups maintained lower P concentrations (~20–40 mg/L), suggesting phosphate depletion potentially due to apatite formation. Although Na levels remained above 3000 mg/L, minimal changes were found, compared to original DMEM, which is abundant in Na^+^ ions. This indicates the negligible impact of BG-derived Na in the medium composition. In contrast, excess ions, especially Ca^2+^ and Si, released by high BG-containing samples could alter osmotic balance or trigger mineral formation, lowering DPSC metabolic activity.

### 3.4. In Vitro Antimicrobial Assays Against E. coli and MRSA

**Figure 5:**
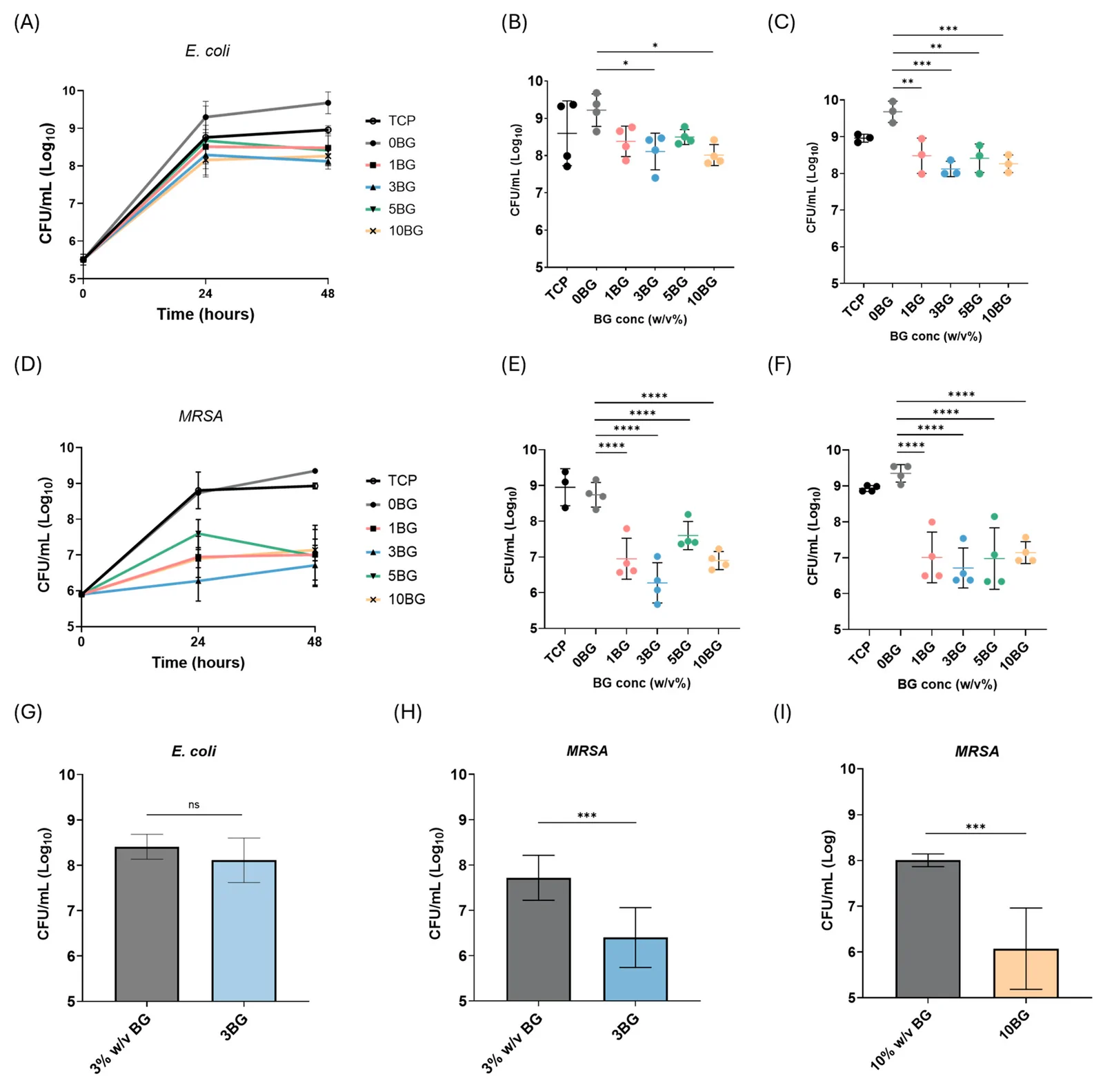
In vitro antibacterial properties of GelMA/BG adhesives against *E. coli* and MRSA. (**A**) Antibacterial time–kill assay curve for *E. coli* in contact with BG-containing hydrogels. (**B**,**C**) CFU counts of *E. coli* after 1 day (**B**) and 2 days (**C**) of incubation with GelMA/BG adhesives. (**D**) Antibacterial time–kill assay curve for MRSA in contact with BG-containing hydrogels. (**E**,**F**) CFU counts of MRSA after 1 day (**E**) and 2 days (**F**) of incubation with GelMA/BG adhesives. (**G**–**I**) Comparison of antibacterial efficacy between free BG particles and GelMA/BG composites at 3 and 10% *w*/*v* BG against *E. coli* (**G**) and MRSA (**H**,**I**), showing enhanced antimicrobial performance from hydrogel-embedded BG particles. Data are presented as the mean ± standard deviation (*n* ≥ 3); not significant (n.s., *p* > 0.05), * (*p* < 0.05), ** (*p* < 0.01), *** (*p* < 0.001), **** (*p* < 0.0001).

The antibacterial activity of GelMA/BG adhesives against *Escherichia coli* (*E. coli*) and methicillin-resistant *Staphylococcus aureus* (MRSA) was evaluated through colony-forming unit (CFU) counting over 2 days (Figure 5A–F). These organisms were used as representative Gram-positive (MRSA) and Gram-negative (*E. coli*) test species to assess antibacterial activity under direct contact. Use of these species enables comparisons with other studies examining the antibacterial activity of BG [38,39]. As demonstrated in Figure 5A, the TCP control and pure GelMA showed rapid bacterial growth, reaching log_10_ CFU/mL values of 8.76 ± 0.84 and 9.30 ± 0.42, respectively, on day 1 and further increasing to 8.96 ± 0.11 and 9.68 ± 0.29 on day 2. In contrast, the BG-containing groups (1, 3, 5 and 10% w/v) significantly suppressed bacterial growth and maintained a log_10_ CFU/mL below 8.7 after 1 day. Notably, 3BG and 10BG achieved 1.00 and 1.14 log reductions, respectively, in *E. coli* CFU/mL, compared to 0BG (Figure 5B). After 2 days, the 0BG group continued to support *E. coli* growth (9.68 ± 0.29 log_10_ CFU/mL), while BG-containing samples continued to suppress bacterial proliferation, with average values ranging from 8.12 to 8.41 log_10_ CFU/mL (Figure 5C). These results showed that *E. coli* suppression by GelMA/BG adhesives was maintained over 2 days.

Likewise, MRSA proliferation was assessed up to 2 days, exhibiting similar BG dose-dependent antibacterial behaviour (Figure 5D). After 1 day, the TCP control and 0BG groups supported substantial MRSA growth; however, BG-containing adhesives significantly inhibited bacterial growth: 6.95 ± 0.57 for 1BG, 6.28 ± 0.56 for 3BG, 7.60 ± 0.39 for 5BG and 6.90 ± 0.25 for 10BG (Figure 5E). GelMA/3BG exhibited the strongest inhibitory function with a 2.46 log reduction compared to the 0BG control on day 1. On day 2, MRSA continued to grow on the 0BG samples, reaching 9.35 ± 0.24 log_10_ CFU/mL, while the addition of BG in all formulations resulted in at least 2 log reductions in MRSA density (Figure 5F). After 2 days, this antibacterial performance was further improved across all BG concentrations. These findings confirmed that increasing BG concentration enhances the antibacterial potential of GelMA hydrogels against both *E. coli* and MRSA, with a stronger and more sustained antibacterial effect against MRSA.

The antibacterial activity of BG particles encapsulated in GelMA matrix was also compared to free BG particles against *E. coli* and MRSA after 1-day incubation. Samples of 3 and 10% *w*/*v* BG were chosen as they were found to have the highest antibacterial properties against *E. coli* and MRSA. Both groups exhibited comparable antimicrobial effects against *E. coli*, with mean log CFU/mL values around 8.4, but the difference was not statistically significant (Figure 5G). Unlike *E. coli*, free 3% *w*/*v* BG particles showed a suppressed MRSA proliferation with a mean log CFU/mL value of 7.72 ± 0.50, whereas the GelMA/3BG composites exhibited a significantly lower density of 6.40 ± 0.66 (Figure 5H). GelMA/10BG also showed the strong inhibitory effect, with nearly a 2-log_10_ reduction, compared to 10% *w*/*v* BG alone (Figure 5I). Overall, both GelMA/3BG and GelMA/10BG hydrogel adhesives significantly enhanced antibacterial efficacy against MRSA compared to BG particles alone.

## 4. Discussion

This study presents an investigation into the incorporation of bioactive glass (BG) particles into gelatin methacryloyl (GelMA) hydrogels, for use as multifunctional dental adhesives. By varying the BG content from 1 to 10% *w*/*v*, we assessed how ion-releasing fillers can modulate the mechanical, adhesive, biological, and antimicrobial properties of the hydrogel matrix. Our findings underscore the potential of GelMA/BG composites to address both mechanical stability and biological compatibility, making them strong candidates for clinical applications in dental tissue regeneration and protection.

The addition of BG significantly influenced the physical properties of the GelMA-based hydrogel adhesives. Under SEM (Figure 1), GelMA exhibited its interconnected porous structure with small pore sizes [40]. The addition of BG particles caused an increase in pore size, except for 10BG, suggesting a more stable structure with high BG concentration. These results are consistent with previous studies reporting the same increasing trend of pore size with lower BG concentrations, which scatters light and interferes with photocrosslinking mechanisms, leading to a slightly reduced crosslinking density [28] (Figure 1A). The microscopic morphologies of hydrogel composites can be strongly influenced by the formation of ice grains during the freeze-drying process, changing pore sizes and original structures [41]. Therefore, we correlated crosslinking density with the observed swelling behaviours, another indicator of structural integrity. The smaller pore size was accompanied by an 18% reduction in the swelling ratio of 10BG, compared to 0BG (Figure 1C). BG encapsulation enhanced structural integrity and reduced water uptake, likely due to ionic interactions between divalent metal ions released from BG particles and the negatively charged functional groups on GelMA chains. In particular, divalent Ca^2+^ ions may form electrostatic bridges between gelatin molecules, serving as secondary physical crosslinks [42]. Furthermore, the binding of Ca^2+^ ions with carboxyl groups reduces the number of free hydrophilic sites available for water absorption, thereby limiting swelling [31]. However, the increased crosslinking density was not reflected in changes to either compressive or tensile modulus at low strains, likely due to the weak, non-covalent interactions between BG particles and the GelMA network, which did not contribute significantly to stiffness [43].

Under large strains, the ultimate tensile strength increased markedly with BG incorporation, with 10BG reaching 96.8 ± 24.9 kPa compared to 58.0 ± 9.5 kPa for 0BG (Figure 1H). These improvements are likely due to filler reinforcement and physical interlocking between BG particles and the polymer matrix [44]. According to Khvostenko et al., BG particles can bridge cracks and bear part of the tensile load, thereby increasing the energy required for crack propagation. This mechanism slows crack progression and helps to resist plastic deformation under large strain. Interestingly, 10% *w*/*v* was confirmed as the optimal and maximal BG content, since higher loading (15BG: 64.5 ± 21.4 kPa) led to reduced mechanical strength. This, however, may result from the destructive effects of highly concentrated Ca^2+^ ions, which destabilise the triple helices of gelatin molecules [45]. Additionally, the observed decline is also likely due to particle agglomeration, which introduces stress concentration sites and disrupts uniform photocrosslinking. Similar behaviour has been reported in PLA/PCL/BG composites, where tensile strength remained stable at lower BG concentrations (5–10 wt%), but a significant reduction occurred at 15 wt% due to particle clustering and impaired stress transfer [46].

In this study, the bioadhesive performance of GelMA and GelMA/BG hydrogels was evaluated on wet collagen sheets under both tensile and lap-shear modes (Figure 2). In tensile adhesion tests, the GelMA precursor was photopolymerised into cylindrical discs directly onto collagen substrates, forming covalent bonds at the interface as reported previously. The incorporation of BG particles significantly enhanced the adhesive strength. Specifically, 10% *w*/*v* BG (10BG) nearly doubled the tensile adhesive strength compared to GelMA alone (54.1 ± 11.2 kPa vs. 27.3 ± 9.0 kPa), whereas excessive BG loading (15% *w*/*v*) resulted in reduced bonding strength (24.9 ± 7.5 kPa). This enhancement is likely due to the alkaline microenvironment and Ca^2+^ ion release induced by BG. During GelMA synthesis, methacrylic acid is formed as a byproduct, lowering the precursor pH to around 4–5, which protonates amino and carboxyl groups on GelMA and tissue surfaces. This protonation hinders free-radical polymerisation and reduces gelation and adhesion efficiency [18]. Upon immersion in fluids, BG particles undergo rapid ion exchange, releasing alkali ions (e.g., Ca^2+^ and Na^+^) and elevating local pH into the slightly alkaline range (pH 7–9), which is more favourable for photocrosslinking [6,47,48]. In addition, Ca^2+^ ions may chelate with carboxyl groups on tissue proteins and GelMA, enhancing interfacial bonding, a mechanism previously reported in alginate-based hydrogels [49]. Also, when GelMA cures, it envelops BG particles and collagen fibrils together, creating mechanical anchorages. Therefore, BG’s presence in the hydrogel could also enhance the physical interlocking with the irregular and porous collagen sheets [50]. In contrast, lap-shear adhesion tests, which simulate shear forces encountered in gingival tissues, showed only modest changes with BG incorporation. This difference likely reflects the fact that BG primarily reinforces the hydrogel bulk to make it more internally rigid, improving resistance to cohesive failure in tensile tests, whereas adhesive failure dominates in lap-shear tests, whereupon the contribution of BG to interfacial strength is more limited [51].

GelMA is derived from collagen and thus inherently interacts well with collagenous surfaces via covalent and non-covalent bonding [52]. However, its adhesion properties were greatly reduced on human tooth sections (Figure 2C) and implant metal plates (Figure 2G), which offer minimal opportunities for chemical bonding or interpenetration. When hydrogel adhesives were photocured on human tooth sections and assayed under tensile adhesion tests, incorporation of BG did not lead to a significant enhancement of adhesive strength as it had previously with collagen sheets. This may reflect the heterogeneous composition of dentine, which consists of both collagen and mineral phases, resulting in reduced exposed collagen available for covalent bonding relative to pure collagen substrates [53]. In addition, the adhesive strength can also be strongly affected by the substrate properties, such as surface tribology, texture and hydrophilicity [54]. Mineralised, relatively inert surfaces are less favourable for GelMA adhesion than collagen-rich substrates. Consistent with this, GelMA-based primers in dentistry have shown comparable bond strength to conventional primers only when dentin is properly acid-etched to expose collagen [52]. Although BG did not enhance immediate bonding to mineralised surfaces, BG promoted HCA formation at the interface, indicating bioactive interaction with dentine.

In addition, lap-shear tests on a Ti6Al4V alloy plate revealed a weaker adhesion of the GelMA-based adhesives, compared to collagen sheets (Figure 2). This outcome is expected, as achieving strong bonding between polymers and metal is challenging due to their surface energy differences and lack of specific interactions [55]. Interestingly, incorporating 10% *w*/*v* BG markedly improved hydrogel adhesion to Ti6Al4V. Titanium alloys are typically covered by a thin, stable oxide layer (primarily TiO_2_) when exposed to air or fluids [56]. The titanium oxide layer could be deprotonated by the high local pH induced by BG upon contact with 10BG adhesives [57]. Ca^2+^ ions sustainably released by BG bridge negatively charged groups on both the GelMA matrix and the negatively charged, deprotonated titanium oxide surface, thus improving the adhesive interaction. Another explanation is that BG particles near the implant surfaces roughen the interface, providing mechanical interlocking sites [49,55]. Over time, it was suggested that mineral nucleation on the TiO_2_ surface is another critical factor to further improve adhesiveness [57]. In summary, under wet conditions, BG was proposed to actively engage in Ca-mediated ionic bonding and promote apatite nucleation, all of which serve to tighten the interface between the GelMA/BG adhesive and a titanium alloy surface.

After the initial adhesion tests, composite–tooth specimens were immersed in DMEM for 7 days to evaluate the long-term effects of in situ mineralisation on bond strength. BG particles alone can form HCA in DMEM after only 3 days (Supplementary Figure S3). Previous studies showed that ion leaching from BG induces the formation of HCA that can bind with collagenous tissues [58]. Consistently, 10BG developed HCA, as confirmed by XRD which showed new apatite peaks on the gel and even sharper peaks on the tooth side of the interface. This suggests mineral bridges (HCA interlocks) formed within the GelMA matrix and at the GelMA–tooth interface. Notably, such mineral bonding would be expected to enhance adhesion. However, an opposing factor is the hydrogel’s swelling in fluid, which can weaken interfacial contact and adhesive strength [59]. It is possible that the benefits of mineral interlocking were offset by hydrogel swelling over 7 days, resulting in no net change in adhesion strength. Together, these results suggest that BG contributes to both hydrogel cohesion and interfacial bonding in wet environments, making GelMA/BG composites promising for soft tissue adhesion and moderately effective on hard dental substrates.

However, BG-induced bioactivity is known to be sensitive to protein-rich environments, as protein adsorption onto BG surfaces in cell culture medium can significantly reduce available surface area and hinder ion exchange [31]. In GelMA hydrogels, BG particles are embedded within a polymer matrix, which not only limits direct exposure of BG surfaces but may also buffer local pH fluctuations, creating a more stable and cell-friendly environment. In this study, live/dead staining showed that GelMA/BG hydrogels maintained high DPSC viability under indirect contact conditions, though the effect was concentration dependent (Figure 3). Although statistically significant differences were observed between 0BG and 3BG, all hydrogel groups exhibited >85% cell viability under indirect contact conditions, meeting high biocompatibility standards [60]. Morphologically, DPSCs retained healthy, elongated fibroblast-like shapes in the 0BG and 1BG groups, whereas increased cell rounding and appearance of dead cells were noted at higher BG concentrations (≥3%). These morphological changes are consistent with previous findings indicating that BG dissolution elevates local pH levels above physiological thresholds (pH > 7.8) within 1 day [8]. Both intracellular and extracellular alkalinisation have been reported to impair cytoskeletal organisation by inducing actin filament depolymerisation, ultimately leading to a rounded morphology [61]. In parallel, elevated pH and Ca^2+^ are known to initiate cellular stress pathways and can lead to cell death, consistent with the cytotoxic thresholds documented in the literature [62,63,64].

Under direct contact, 1BG significantly promoted DPSC proliferation compared to 0BG. In contrast, higher BG concentrations (≥3% *w*/*v* BG) suppressed metabolic activity. These effects were most pronounced during the first day, correlating with pH elevation and ion release behaviour [65]. While culture medium pH remained relatively stable in all BG-containing groups (~8.2–8.4), the control (0BG) maintained a slightly lower pH (~8.0) (Figure 4). The alkaline shift is characteristic of BG dissolution, driven by ion exchange of Na^+^ and Ca^2+^ ions for H^+^ from the surrounding solution, leading to proton consumption and pH elevation [8]. Despite appearing modest in bulk measurements, even subtle increases in medium pH have been reported to significantly reduce DPSC metabolism. This may be explained by the concept of interfacial pH, the local pH at the hydrogel–cell interface, which can differ substantially from the bulk medium [66]. Liu et al. demonstrated bioactive glass surfaces exhibited a significantly higher interfacial pH than the surrounding blood [67]. Particularly, cells are highly sensitive to these local microenvironmental changes; even small alkaline shifts can deprotonate amino acid side chains, disrupt adhesion proteins and impair cytoskeletal stability, ultimately hindering adhesion and proliferation [68]. Notably, the GelMA matrix serves a buffering role, preconditioning the BG particles and mitigating the initial pH surge during early dissolution, thereby reducing the potential cytotoxic effects [63].

Regarding Ca^2+^ dynamics, an initial rapid decline in calcium concentration was observed, likely due to cellular uptake, HCA formation and adsorption to the gelatin-based hydrogel matrix, as previously discussed in gelatin and alginate systems [45,49]. After 4 days, continued BG dissolution saturated the GelMA matrix, leading to Ca^2+^ leaching into the medium and stabilising extracellular calcium at 60–80 ppm. Moderate extracellular Ca^2+^ concentrations within this range have been shown to enhance DPSC proliferation and osteogenic differentiation through MAPK and Wnt/β-catenin signalling pathways [69,70]. A secondary decline in calcium levels was detected by day 7, which may be attributed to calcium phosphate and apatite mineral deposition, aligning with concurrent phosphate and silicon ion depletion observed in the medium. Soluble silica species (~60–70 ppm) were steadily released and are known to enhance DPSC proliferation and early osteogenic commitment, both independently and synergistically with Ca^2+^ ions [70]. In contrast, although BG releases Na^+^ through ionic exchange, the high baseline sodium concentration in DMEM (~3000 mg/L) rendered the additional contribution from BG negligible in altering media composition. Despite the initial suppression of metabolic activity, DPSCs exposed to BG-loading hydrogels showed signs of recovery by day 4, indicating a transient stress response followed by partial adaptation to the BG-dissolution environment [71].

Thanks to its alkaline nature and ionic dissolution products, 45S5 bioactive glass exhibits broad-spectrum antibacterial activity [72]. Upon dissolution, BG releases Na^+^, Ca^2+^ and other ions that increase extracellular osmolarity and disrupt bacterial membrane potential, particularly when ion concentrations rise rapidly [73]. In a study by Hu et al., 45S5 BG particles demonstrated potent antimicrobial effects against *S. aureus*, *S. epidermidis* and *E. coli* at concentrations ≥ 50 mg/mL, indicating a dose-dependent response. Notably, they found that direct contact with BG particles was more bactericidal than ionic extracts alone, even under comparable alkaline conditions (~pH 9.8), highlighting the role of physical interactions. TEM imaging revealed needle-like BG debris in damaged bacterial membranes, suggesting mechanical disruption as an additional mechanism of action. Interestingly, *E. coli* appeared more sensitive than Gram-positive species in their study, a trend that contrasts with our findings.

In the present study, the antibacterial properties of GelMA/BG composites were assessed against *E. coli* and MRSA over 2 days (Figure 5). BG incorporation significantly reduced bacterial counts in both species relative to the control. The composites suppressed bacteria proliferation compared to GelMA alone. Notably, antibacterial therapies do not necessarily sterilise the relevant site but rather reduce bacterial growth to a level that can be cleared or tolerated by host defences [74]. For example, several highly efficacious antibiotics, such as tetracyclines, are bacteriostatic, inhibiting bacterial growth rather than directly killing bacteria [75].

Here, the inhibitory effect was more pronounced against MRSA. This discrepancy likely reflects differences in bacterial adhesion behaviours. Gram-positive bacteria, including MRSA, possess a more hydrophobic cell surface, which promotes stronger attachment to biomaterials [76]. MRSA is also known to adhere to both hydrophilic and hydrophobic substrates and expresses fibronectin proteins that facilitate strong interactions with gelatin-based hydrogels [77,78]. As a result, MRSA is more likely to remain in contact with the adhesive surface, experiencing localised ionic and pH perturbations, which may amplify antibacterial effects.

In contrast, *E. coli* tends to remain planktonic and exhibits weaker interactions with protein-based hydrogels, particularly under conditions of low stiffness [79]. Furthermore, as a Gram-negative species, *E. coli* has an additional outer membrane that provides enhanced protection against environmental stress, including mild alkalinity. This structural barrier, combined with Na^+^/H^+^ antiporters that regulate intracellular pH, enables *E. coli* to better tolerate alkaline conditions [80]. Nevertheless, BG-containing hydrogels still achieved stable suppression of *E. coli* over 2 days, suggesting that even Gram-negative bacteria are susceptible to prolonged ionic and osmotic stress when BG is effectively retained and activated within the hydrogel.

Importantly, all GelMA/BG groups maintained or slightly improved antibacterial efficacy over 2 days, with no regrowth in bacterial population. This sustained suppression highlights a key advantage of the composite system: the GelMA matrix not only buffers abrupt pH shifts but also retains BG particles at the interface, enabling continued ion release. Ion release from the glass into the gel is likely to have started before immersion into the bacteria broth. This preconditioning likely raises the early pH threshold at the hydrogel–bacteria interface, allowing sustained antibacterial activity against surrounding bacteria and contributing to the enhanced efficacy compared with free BG particles [72,81]. Thus, despite its buffering capacity, GelMA may synergise with BG to potentiate antibacterial action in a clinically relevant setting following mechanical plaque removal. Following mechanical debridement, the GelMA/BG adhesives could be applied to limit bacterial recolonisation and prevent re-establishment of plaque within the periodontal pocket [82,83]. Sustained bacterial suppression is particularly relevant in the oral cavity, where the microbial burden is inherently high and complete bacterial eradication is impossible [84]. Future studies using complex oral biofilms and additional periodontal pathogens, such as *P. gingivalis*, would further establish the clinical relevance of these findings.

Beyond its intrinsic antibacterial activity, the GelMA/BG adhesives could also be used alongside other periodontal treatments rather than as a standalone antibacterial therapy [85]. This may provide an additional advantage when combined with antimicrobial treatment, as the alkaline environment generated by BG dissolution could enhance the efficacy of certain antibiotics administered alongside the adhesive [86]. A recent systematic review identified that several antibiotics, such as fluoroquinolones, aminoglycosides and trimethoprim, exhibit stronger activity at alkaline pH levels compared to acidic or neutral environments [87]. In support, studies have demonstrated that raising extracellular pH can potentiate the killing activity of gentamicin against both Gram-positive and Gram-negative bacteria [88]. This suggests that the GelMA/BG adhesives could work in synergy with antibiotics that perform better at higher pH levels, such as gentamicin, to accelerate bacterial killing, especially in persistent biofilm infections commonly found in periodontal disease.

Combining the adhesive with appropriate antibiotics could potentially reduce the required antibiotic dosage, shorten treatment duration and help to reduce the likelihood of resistance development. Importantly, this strategy leverages the adhesive’s inherent, non-antibiotic antimicrobial action, while using antibiotics more effectively thanks to the locally elevated pH environment created by BG dissolution.

## 5. Conclusions

In summary, we developed a multifunctional GelMA/BG hydrogel adhesive that forms robust bonds to moist collagen-rich tissues through visible-light crosslinking. The composite demonstrated durable mechanical adhesion, cytocompatibility with dental pulp stem cells and dose-dependent antimicrobial activity against *E. coli* and MRSA, in terms of suppressing their proliferation compared to the control due to the embedding of BG particles in the gels. These combined properties address key challenges in periodontal therapy, achieving both physical sealing and biological protection of affected sites. Given its promising in vitro performance, the GelMA/BG composite is a strong candidate for next-generation periodontal or peri-implant applications.

## Data Availability

The data that supports the findings of this study are available from at 10.5281/zenodo.20590989. For the purpose of open access, the authors have applied a Creative Commons Attribution (CC BY) license (where permitted by UKRI, ‘Open Government License’).

## Supplementary Materials

The following supporting information can be downloaded at: https://www.mdpi.com/article/10.3390/jfb17080394/s1, Figure S1. Particle size distribution of 45S5 BG particles measured by laser diffraction; Figure S2. XRD pattern of 45S5 BG particles, confirming the amorphous structure of the glass; Figure S3. XRD pattern of 45S5 BG particles after immersion in DMEM for 3 days, showing hydroxycarbonate apatite peaks (ICCD reference code: 00-009-0432) [29]; Figure S4. 1H NMR spectra of 3% *w*/*v* GelMA and gelatin in D2O, adapted from Zhu et al. [18]; Figure S5. Morphology of GelMA and GelMA/BG composites of 3% *w*/*v* BG; Figure S6. Cumulative elemental profiles of Ca, Si, P and Na in cell culture media from GelMA/BG adhesives with 0, 1, 3, 5 and 10% *w*/*v* BG, measured using ICP-OES.

## References

[1] Dentino A., Lee S., Mailhot J., Hefti A.F. (2013). Principles of periodontology. Periodontology 2000.

[2] Edwards P.C., Kanjirath P. (2010). Recognition and management of common acute conditions of the oral cavity resulting from tooth decay, periodontal disease, and trauma: An update for the family physician. J. Am. Board Fam. Med..

[3] Lee F.Y., Chen D.W., Hu C.C., Hsieh Y.T., Liu S.J., Chan E.C. (2011). In vitro and in vivo investigation of drug-eluting implants for the treatment of periodontal disease. AAPS PharmSciTech.

[4] Page R.C. (1998). The Pathobiology of Periodontal Diseases May Affect Systemic Diseases: Inversion of a Paradigm. Ann. Periodontol..

[5] Goodson J.M. (1994). Antimicrobial strategies for treatment of periodontal diseases. Periodontology 2000.

[6] Jones J.R. (2013). Review of bioactive glass: From Hench to hybrids. Acta Biomater..

[7] Skallevold H.E., Rokaya D., Khurshid Z., Zafar M.S. (2019). Bioactive Glass Applications in Dentistry. Int. J. Mol. Sci..

[8] Gholami S., Labbaf S., Houreh A.B., Ting H.K., Jones J.R., Esfahani M.H.N. (2017). Long term effects of bioactive glass particulates on dental pulp stem cells in vitro. Biomed. Glasses.

[9] Xynos I.D., Edgar A.J., Buttery L.D.K., Hench L.L., Polak J.M. (2001). Gene-expression profiling of human osteoblasts following treatment with the ionic products of Bioglass^®^ 45S5 dissolution. J. Biomed. Mater. Res..

[10] Apel C., Buttler P., Salber J., Dhanasingh A., Neuss S. (2018). Differential mineralization of human dental pulp stem cells on diverse polymers. Biomed. Tech..

[11] Lazzeri S., Montagnani C., Zanardi A., Beltrami G., Galli L. (2022). Bioactive glass in the treatment of chronic osteomyelitis in children: Description of four consecutive cases and literature review. Injury.

[12] Zhou P., Garcia B.L., Kotsakis G.A. (2022). Comparison of antibacterial and antibiofilm activity of bioactive glass compounds S53P4 and 45S5. BMC Microbiol..

[13] Dong Z., Sun Y., Chen Y., Liu Y., Tang C., Qu X. (2019). Injectable Adhesive Hydrogel through a Microcapsule Cross-Link for Periodontitis Treatment. ACS Appl. Bio Mater..

[14] Yue K., Trujillo-de Santiago G., Alvarez M.M., Tamayol A., Annabi N., Khademhosseini A. (2015). Synthesis, properties, and biomedical applications of gelatin methacryloyl (GelMA) hydrogels. Biomaterials.

[15] Nichol J.W., Koshy S.T., Bae H., Hwang C.M., Yamanlar S., Khademhosseini A. (2010). Cell-laden microengineered gelatin methacrylate hydrogels. Biomaterials.

[16] Liao J., Qiu J., Lin Y., Li Z. (2024). The application of hydrogels for enamel remineralization. Heliyon.

[17] Loessner D., Meinert C., Kaemmerer E., Martine L.C., Yue K., Levett P.A., Klein T.J., Melchels F.P.W., Khademhosseini A., Hutmacher D.W. (2016). Functionalization, preparation and use of cell-laden gelatin methacryloyl-based hydrogels as modular tissue culture platforms. Nat. Protoc..

[18] Zhu M., Wang Y., Ferracci G., Zheng J., Cho N.J., Lee B.H. (2019). Gelatin methacryloyl and its hydrogels with an exceptional degree of controllability and batch-to-batch consistency. Sci. Rep..

[19] Bertlein S., Brown G., Lim K.S., Jungst T., Boeck T., Blunk T., Tessmar J., Hooper G.J., Woodfield T.B.F., Groll J. (2017). Thiol–Ene Clickable Gelatin: A Platform Bioink for Multiple 3D Biofabrication Technologies. Adv. Mater..

[20] Greene T., Lin T., Andrisani O.M., Lin C. (2017). Comparative study of visible light polymerized gelatin hydrogels for 3D culture of hepatic progenitor cells. J. Appl. Polym. Sci..

[21] Lim K.S., Schon B.S., Mekhileri N.V., Brown G.C.J., Chia C.M., Prabakar S., Hooper G.J., Woodfield T.B.F. (2016). New Visible-Light Photoinitiating System for Improved Print Fidelity in Gelatin-Based Bioinks. ACS Biomater. Sci. Eng..

[22] Elvin C.M., Carr A.G., Huson M.G., Maxwell J.M., Pearson R.D., Vuocolo T., Brownlee A.G., McFarland G.A., McKimmie C.S., Taylor K.M. (2010). A highly elastic tissue sealant based on photopolymerised gelatin. Biomaterials.

[23] Lim K.S., Kurniawan N.A., Chia C.M., Meagher L., O’Connor A.J., Woodruff M.A., Bock N., Woodfield T.B.F. (2019). Visible Light Cross-Linking of Gelatin Hydrogels Offers an Enhanced Cell Microenvironment with Improved Light Penetration Depth. Macromol. Biosci..

[24] Yu L., Chen Y., Liu Y., Wang Y., Zhang H., Li J., Zhao X., Wang S., Li X., Zhang Y. (2024). Sequential-Crosslinking Fibrin Glue for Rapid and Reinforced Hemostasis. Adv. Sci..

[25] Daniele M.A., Adams A.A., Naciri J., North S.H., Ligler F.S. (2014). Interpenetrating networks based on gelatin methacrylamide and PEG formed using concurrent thiol click chemistries for hydrogel tissue engineering scaffolds. Biomaterials.

[26] Yang-Jensen K.C., Jørgensen S.M., Chuang C.Y., Davies M.J. (2024). Modification of extracellular matrix proteins by oxidants and electrophiles. Biochem. Soc. Trans..

[27] Chen S., Liu M., Wang X., Zhang Y., Zhou Y., Chen Y., Li J., Wang H., Li X. (2021). Adhesive Tissue Engineered Scaffolds: Mechanisms and Applications. Front. Bioeng. Biotechnol..

[28] Zheng J., Chen X., Zhang X., Wang Y., Liu Y., Chen H., Chen Y., Li X. (2018). Sequentially-crosslinked biomimetic bioactive glass/gelatin methacryloyl composites hydrogels for bone regeneration. Mater. Sci. Eng. C.

[29] Jalilinejad N., Baheiraei N., Azami M., Ramezani M., Haramshahi S.M.A., Shahrezaee M. (2025). Fabrication and characterizations of 3D printed GelMA-Gel/bioactive glass scaffolds containing cerium for bone damage repair. Sci. Rep..

[30] Chen H., Chen Y., He H., Zhou X., Chen N. (2023). Synthesis of Silanized Bioactive Glass/Gelatin Methacrylate (GelMA/Si-BG) composite hydrogel for Bone Tissue Engineering Application. J. Mech. Behav. Biomed. Mater..

[31] Akhtar M., Peng P., Bernhardt A., Gelinsky M., Ur Rehman M.A., Boccaccini A.R., Basu B. (2024). Gelatin Methacryloyl (GelMA)-45S5 Bioactive Glass (BG) Composites for Bone Tissue Engineering: 3D Extrusion Printability and Cytocompatibility Assessment Using Human Osteoblasts. ACS Biomater. Sci. Eng..

[32] Mei N., Li Y., Chen Y., Zhang H., Wang X., Li J., Wang Y. (2022). 3D-printed mesoporous bioactive glass/GelMA biomimetic scaffolds for osteogenic/cementogenic differentiation of periodontal ligament cells. Front. Bioeng. Biotechnol..

[33] Ghorbani F., Sahranavard M., Nejad Z.M., Li D., Zamanian A., Yu B. (2020). Surface Functionalization of Three Dimensional-Printed Polycaprolactone-Bioactive Glass Scaffolds by Grafting GelMA Under UV Irradiation. Front. Mater..

[34] Jayawardena I., Turunen P., Garms B.C., Rowan A., Corrie S., Grøndahl L. (2023). Evaluation of techniques used for visualisation of hydrogel morphology and determination of pore size distributions. Mater. Adv..

[35] Duta L., Stan G.E., Popescu-Pelin G., Zgura I., Anastasescu M., Oktar F.N. (2022). Influence of Post-Deposition Thermal Treatments on the Morpho-Structural, and Bonding Strength Characteristics of Lithium-Doped Biological-Derived Hydroxyapatite Coatings. Coatings.

[36] (2009). Biological Evaluation of Medical Devices, Part 5: Tests for In Vitro Cytotoxicity, 3rd ed.

[37] (2021). Biological Evaluation of Medical Devices, Part 12: Sample Preparation and Reference Materials.

[38] Wu D., Wang P., Wu Q., Chu C.H., Lei C., Wu W., Ma S., Lv J., Tang C. (2022). Preparation and characterization of Bomidin-loaded thermosensitive hydrogel for periodontal application. J. Mater. Res..

[39] Zhu X., Xiu X., Jiang X., Wang Y., Li J., Zhang H., Liu Y., Chen X., Zhou L., Wang Z. (2025). Injectable hydrogels with ROS-triggered drug release enable the co-delivery of antibacterial agent and anti-inflammatory nanoparticle for periodontitis treatment. J. Nanobiotechnol..

[40] Yin H., Zhu M., Wang Y., Luo L., Ye Q., Lee B.H. (2023). Physical properties and cellular responses of gelatin methacryloyl bulk hydrogels and highly ordered porous hydrogels. Front. Soft Matter.

[41] Grenier J., Duval H., Barou F., Lv P., David B., Letourneur D. (2019). Mechanisms of pore formation in hydrogel scaffolds textured by freeze-drying. Acta Biomater..

[42] Chatterjee S., Bohidar H.B. (2005). Effect of cationic size on gelation temperature and properties of gelatin hydrogels. Int. J. Biol. Macromol..

[43] Oosterbeek R.N., Zhang X.C., Best S.M., Cameron R.E. (2022). The evolution of the structure and mechanical properties of fully bioresorbable polymer-glass composites during degradation. Compos. Sci. Technol..

[44] Khvostenko D., Mitchell J.C., Hilton T.J., Ferracane J.L., Kruzic J.J. (2013). Mechanical performance of novel bioactive glass containing dental restorative composites. Dent. Mater..

[45] Naolou T., Schadzek N., Hornbostel J.M., Pepelanova I., Frommer M., Lötz F., Sauheitl L., Dultz S., Felde V.J.M.N.L., Myklebost O. (2025). Enhanced gelatin methacryloyl nanohydroxyapatite hydrogel for high-fidelity 3D printing of bone tissue engineering scaffolds. Biofabrication.

[46] Chen M., Chen Y., He H., Zhou X., Chen N. (2025). Structure and Property Evolution of Microinjection Molded PLA/PCL/Bioactive Glass Composite. Polymers.

[47] Abete T., Del Gado E., de Arcangelis L., Serughetti D.H., Djabourov M. (2008). Reentrant phase diagram and pH effects in cross-linked gelatin gels. J. Chem. Phys..

[48] Valdebenito A., Encinas M.V. (2003). Photopolymerization of 2-hydroxyethyl methacrylate: Effect of the medium properties on the polymerization rate. J. Polym. Sci. A Polym. Chem..

[49] Gao L., Zhou Y., Peng J., Xu C., Xu Q., Xing M., Chang J. (2019). A novel dual-adhesive and bioactive hydrogel activated by bioglass for wound healing. NPG Asia Mater..

[50] Ledesma E., Rendueles M., Díaz M. (2015). Characterization of natural and synthetic casings and mechanism of BaP penetration in smoked meat products. Food Control.

[51] Barreto M.E.V., Medeiros R.P., Shearer A., Fook M.V.L., Montazerian M., Mauro J.C. (2023). Gelatin and Bioactive Glass Composites for Tissue Engineering: A Review. J. Funct. Biomater..

[52] Alia A., Gao F., Mitchell J.C., Gasiorowski J., Ciancio M., Kuppast B., Pfeifer C., Carrilho M.R. (2023). Dentin primer based on a highly functionalized gelatin-methacryloyl hydrogel. Dent. Mater..

[53] Saikaew P., Sattabanasuk V., Harnirattisai C., Chowdhury A.F.M.A., Carvalho R., Sano H. (2022). Role of the smear layer in adhesive dentistry and the clinical applications to improve bonding performance. Jpn. Dent. Sci. Rev..

[54] Pal S., Peršin Z., Vuherer T., Drstvenšek I., Kokol V. (2020). The effect of Ti-6Al-4V alloy surface structure on the adhesion and morphology of unidirectional freeze-coated gelatin. Coatings.

[55] Yamaguchi S., Akeda K., Shintani S.A., Sudo A., Matsushita T. (2022). Drug-Releasing Gelatin Coating Reinforced with Calcium Titanate Formed on Ti–6Al–4V Alloy Designed for Osteoporosis Bone Repair. Coatings.

[56] Liang J., Lu X., Zheng X., Li Y.R., Geng X., Sun K., Cai H., Jia Q., Jiang H.B., Liu K. (2023). Modification of titanium orthopedic implants with bioactive glass: A systematic review of in vivo and in vitro studies. Front. Bioeng. Biotechnol..

[57] Ajami E., Aguey-Zinsou K.-F. (2012). Calcium Phosphate Growth at Electropolished Titanium Surfaces. J. Funct. Biomater..

[58] Kazem N.E., El-Refai D.A., Alian G. (2024). Assessment of physical properties of bioactive glass-modified universal multimode adhesive and its bonding potential to artificially induced caries affected dentin. BMC Oral Health.

[59] Tian G., Yang D., Liang C., Liu Y., Chen J., Zhao Q., Tang S., Huang J., Xu P., Liu Z. (2023). A Nonswelling Hydrogel with Regenerable High Wet Tissue Adhesion for Bioelectronics. Adv. Mater..

[60] Nandhakumar M., Thangaian D.T., Sundaram S., Roy A., Subramanian B. (2022). An enduring in vitro wound healing phase recipient by bioactive glass-graphene oxide nanocomposites. Sci. Rep..

[61] Stock C. (2024). pH-regulated single cell migration. Pflüg. Arch. Eur. J. Physiol..

[62] Ciraldo F.E., Boccardi E., Melli V., Westhauser F., Boccaccini A.R. (2018). Tackling bioactive glass excessive in vitro bioreactivity: Preconditioning approaches for cell culture tests. Acta Biomater..

[63] Hohenbild F., Arango-Ospina M., Moghaddam A., Boccaccini A.R., Westhauser F. (2020). Preconditioning of bioactive glasses before introduction to static cell culture: What is really necessary?. Methods Protoc..

[64] Silver I.A., Deas J., Erecinh M. (2001). Interactions of bioactive glasses with osteoblasts in vitro: Effects of 45S5 Bioglass, and 58S and 77S bioactive glasses on metabolism, intracellular ion concentrations and cell viability. Biomaterials.

[65] Labbaf S., Tsigkou O., Müller K.H., Stevens M.M., Porter A.E., Jones J.R. (2011). Spherical bioactive glass particles and their interaction with human mesenchymal stem cells in vitro. Biomaterials.

[66] Ruan C., Hu N., Ma Y., Li Y., Liu J., Zhang X., Pan H. (2017). The interfacial pH of acidic degradable polymeric biomaterials and its effects on osteoblast behavior. Sci. Rep..

[67] Liu W., Li J., Cheng M., Wang Q., Zhang X., Zhang Z., Wang Y. (2016). Alkaline biodegradable implants for osteoporotic bone defects—Importance of microenvironment pH. Osteoporos. Int..

[68] Beaumont K.G., Mrksich M. (2012). The Mechanostability of Isolated Focal Adhesions Is Strongly Dependent on pH. Chem. Biol..

[69] Maeno S., Niki Y., Matsumoto H., Morioka H., Yatani Y., Toyama Y. (2005). The effect of calcium ion concentration on osteoblast viability, proliferation and differentiation in monolayer and 3D culture. Biomaterials.

[70] Tousi N.S., Velten M.F., Bishop P., Bonfield W., Boccaccini A.R. (2013). Combinatorial effect of Si^4+^, Ca^2+^, and Mg^2+^ released from bioactive glasses on osteoblast osteocalcin expression and biomineralization. Mater. Sci. Eng. C.

[71] Deb S., Mandegaran R., Di Silvio L. (2010). A porous scaffold for bone tissue engineering/45S5 Bioglass^®^ derived porous scaffolds for co-culturing osteoblasts and endothelial cells. J. Mater. Sci. Mater. Med..

[72] Munukka E., Leppäranta O., Korkeamäki M., Vaahtio M., Peltola T., Zhang D., Hupa L., Ylänen H., Salonen J.I., Viljanen M.K. (2008). Bactericidal effects of bioactive glasses on clinically important aerobic bacteria. J. Mater. Sci. Mater. Med..

[73] Drago L., Toscano M., Bottagisio M. (2018). Recent evidence on bioactive glass antimicrobial and antibiofilm activity: A mini-review. Materials.

[74] Marsh P.D. (2006). Dental plaque as a biofilm and a microbial community: Implications for health and disease. BMC Oral Health.

[75] Bunick C.G., Keri J., Tanaka S.K., Furey N., Damiani G., Johnson J.L., Grada A. (2021). Antibacterial mechanisms and efficacy of sarecycline in animal models of infection and inflammation. Antibiotics.

[76] Krasowska A., Sigler K. (2014). How microorganisms use hydrophobicity and what does this mean for human needs?. Front. Cell. Infect. Microbiol..

[77] Castonguay M.H., van der Schaaf S., Koester W., Krooneman J., van der Meer W., Harmsen H., Landini P. (2006). Biofilm formation by Escherichia coli is stimulated by synergistic interactions and co-adhesion mechanisms with adherence-proficient bacteria. Res. Microbiol..

[78] Arciola C.R., Campoccia D., Montanaro L. (2018). Implant infections: Adhesion, biofilm formation and immune evasion. Nat. Rev. Microbiol..

[79] Kolewe K.W., Peyton S.R., Schiffman J.D. (2015). Fewer Bacteria Adhere to Softer Hydrogels. ACS Appl. Mater. Interfaces.

[80] Padan E., Bibi E., Ito M., Krulwich T.A. (2005). Alkaline pH homeostasis in bacteria: New insights. Biochim. Biophys. Acta Biomembr..

[81] Fu M., Liang Y., Lv X., Li C., Yang Y.Y., Yuan P., Ding X. (2023). Recent advances in hydrogel-based anti-infective coatings. J. Mater. Sci. Technol..

[82] Shah V., Shetty P., Shetty D., Masurkar D., Kshirsagar Y., Agrawal K., Almalki S.A., Alqahtani A.S., Alsakr A., Gufran K. (2026). Effectiveness of liquorice gel as an adjunct to non-surgical periodontal therapy in chronic periodontitis: A randomized controlled, clinical and microbiological trial. Sci. Rep..

[83] Nudrath A., Bodduru R.R., Boyapati R., Hassan S., Hari S., Vadla A. (2026). Comparative efficacy of a local drug delivery system using Blue^®^M gel and Gengigel^®^ as an adjunct to scaling and root planing in chronic periodontitis patients. Dent. Med. Probl..

[84] Darveau R. (2010). Periodontitis: A polymicrobial disruption of host homeostasis. Nat. Rev. Microbiol..

[85] Khademi R., Hosseini M.A., Kharaziha M. (2025). An injectable gelatin methacrylate containing surface-imprinted chitosan-modified bioglass microspheres for potential periodontitis treatment. Int. J. Biol. Macromol..

[86] Fan M., Ren Y., Zhu Y., Zhang H., Li S., Liu C., Lv H., Chu L., Hou Z., Zhang Y. (2025). Borosilicate bioactive glass synergizing low-dose antibiotic loaded implants to combat bacteria through ATP disruption and oxidative stress to sequentially achieve osseointegration. Bioact. Mater..

[87] Ordaz G., Dagà U., Budia A., Pérez-Lanzac A., Fernández J.M., Jordán C. (2023). Urinary pH and antibiotics, choose carefully. A systematic review. Actas Urol. Esp..

[88] Zhu C., Zhou Y., Kang J., Yang H., Lin J., Fang B. (2023). Alkaline arginine promotes the gentamicin-mediated killing of drug-resistant Salmonella by increasing NADH concentration and proton motive force. Front. Microbiol..

