# Supplementary material for: Antimicrobial Gelatin Methacryloyl (GelMA)/Bioactive Glass Dental Adhesives
Source: J Funct Biomater. 2026 Aug 10;17(8):394. doi: 10.3390/jfb17080394 (PMC13514707; doi:10.3390/jfb17080394)
Supplement: Supplementary file 1 [file jfb-17-00394-s001.zip › jfb-4395484-supplementary.pdf]

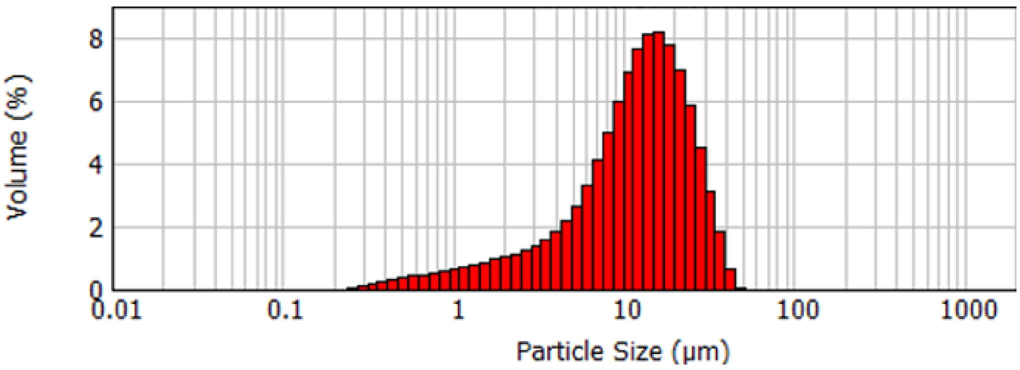

Figure S1. Particle size distribution of 45S5 BG particles measured by laser diffraction.

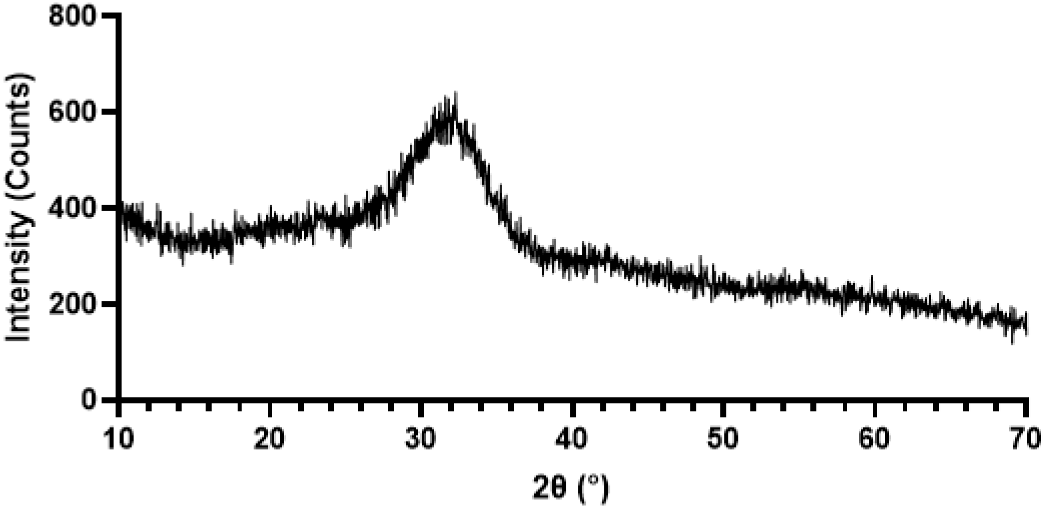

Figure S2. XRD pattern of 45S5 BG particles, confirming the amorphous structure of the glass.

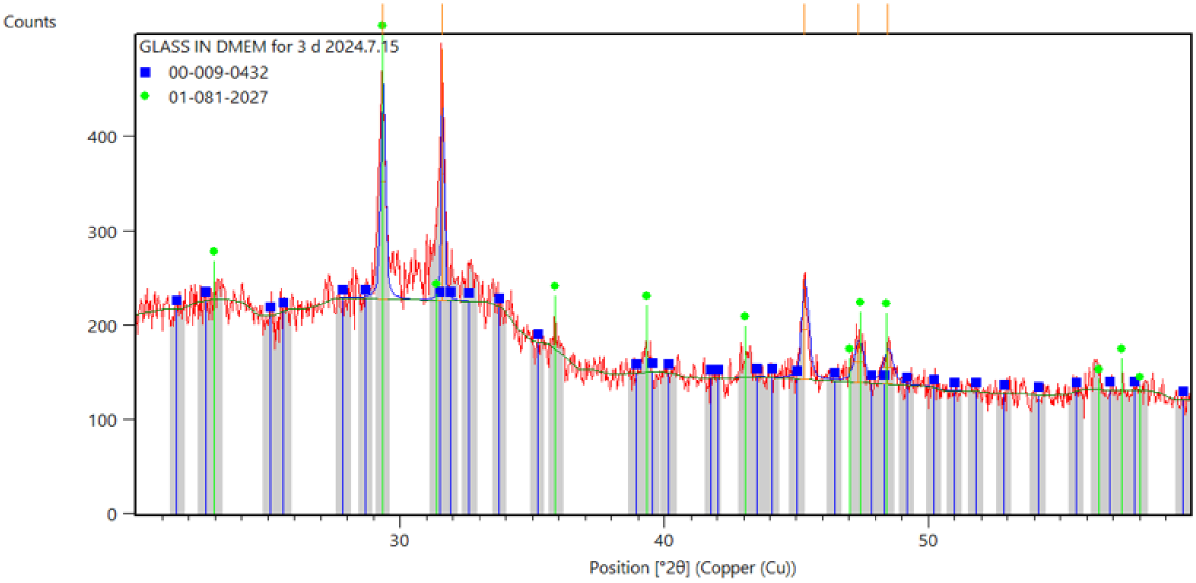

Figure S3. XRD pattern of 45S5 BG particles after immersion in DMEM for 3 days, showing hydroxycarbonate apatite peaks (ICCD reference code: 00-009-0432) [29].

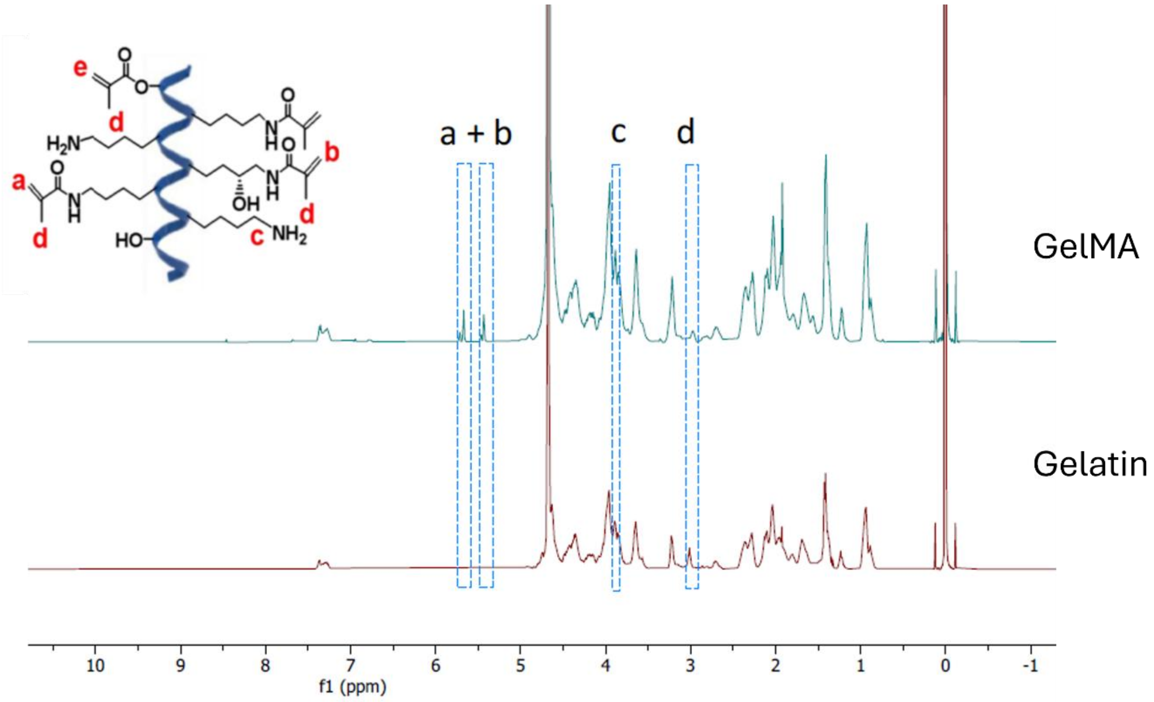

Figure S4. ^{1}H NMR spectra of 3% w/v GelMA and gelatin in D_2O, adapted from Zhu *et al.* [18].

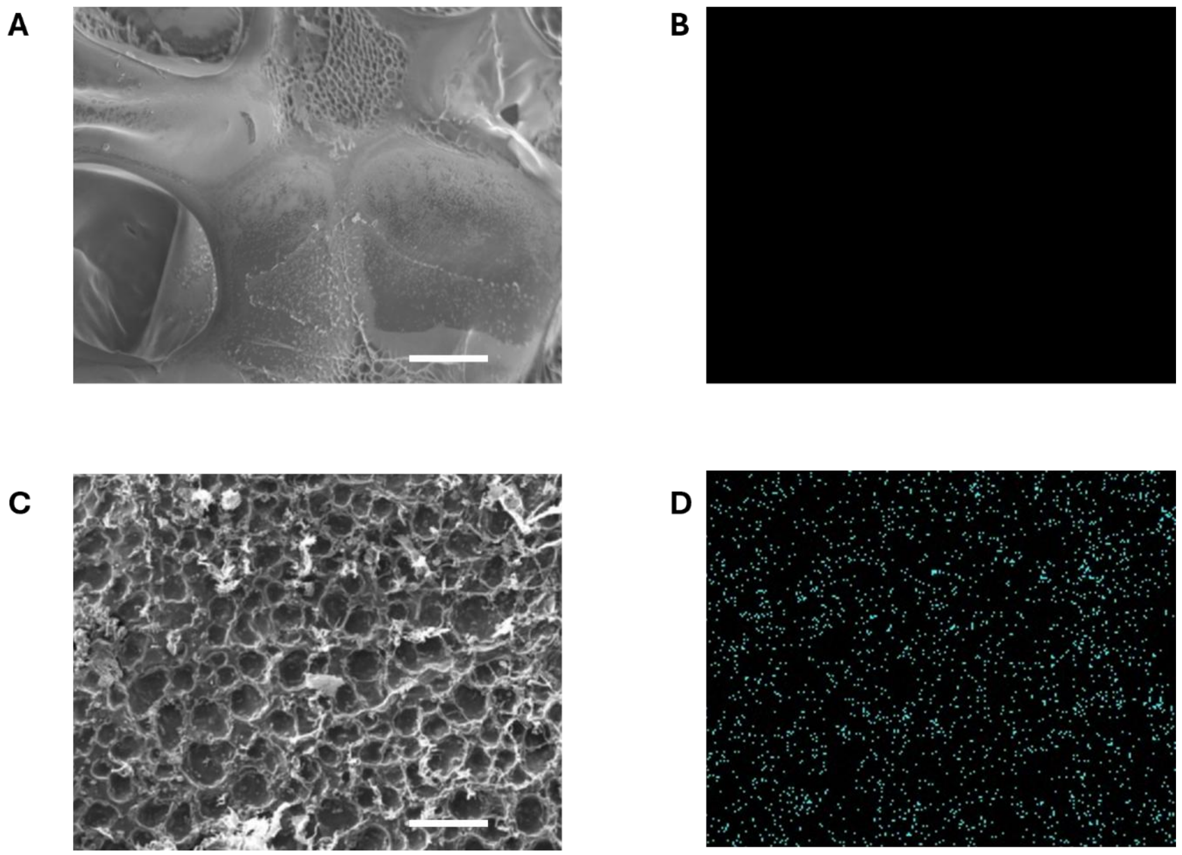

Figure S5. Morphology of GelMA and GelMA/BG composites of 3% w/v BG. (A) SEM micrograph of GelMA; (B) EDX Si map of GelMA; (C) SEM of GelMA/BG; (D) EDX Si map of GelMA/BG. Scale bar = 100 µm.

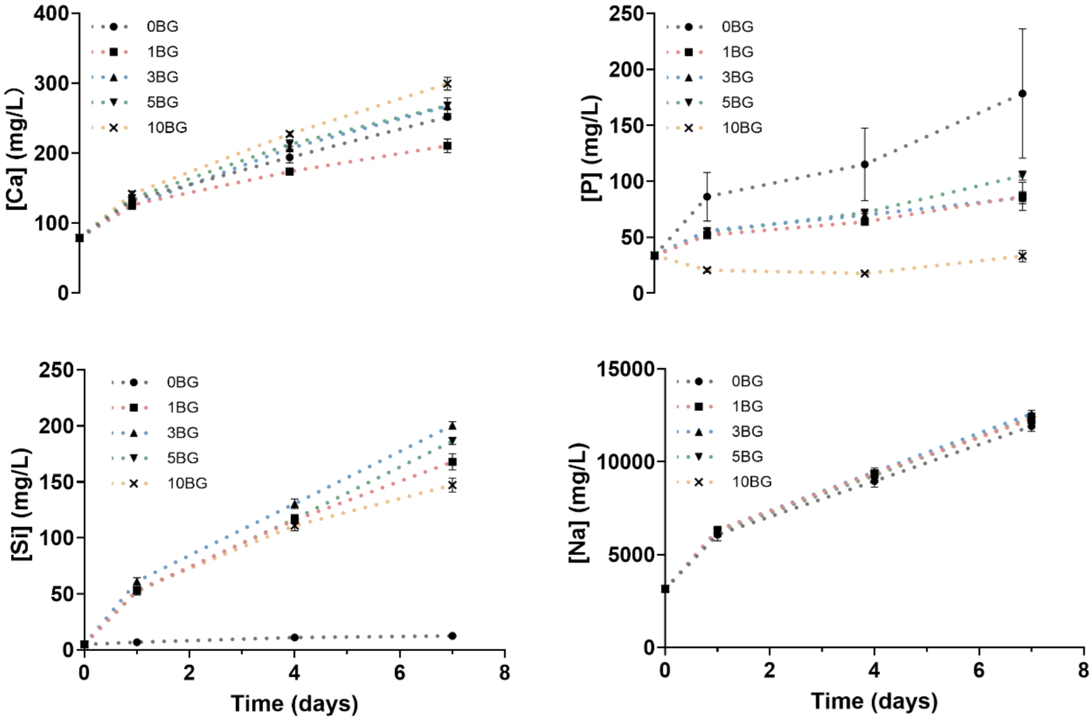

Figure S6. Cumulative elemental profiles of Ca, Si, P and Na in cell culture media from GelMA/BG adhesives with 0, 1, 3, 5 and 10% w/v BG, measured using ICP-OES.
